# BEACH domain proteins function as cargo-sorting adaptors in secretory and endocytic pathways

**DOI:** 10.1083/jcb.202408173

**Published:** 2024-11-08

**Authors:** Serhiy Pankiv, Anette Kathinka Dahl, Aleksander Aas, Rosa Linn Andersen, Andreas Brech, Petter Holland, Sakshi Singh, Christian Bindesbøll, Anne Simonsen

**Affiliations:** 1 Department of Molecular Medicine, Institute of Basic Medical Sciences, University of Oslo, Oslo, Norway; 2 Centre for Cancer Cell Reprogramming, Faculty of Medicine, University of Oslo, Oslo, Norway; 3 Department of Molecular Cell Biology, Institute for Cancer Research, Oslo University Hospital, Oslo, Norway; 4 Department of Core Facilities, Institute for Cancer Research, Oslo University Hospital, Oslo, Norway

## Abstract

We identify BEACH domain–containing proteins (BDCPs) as novel membrane coat proteins involved in the sorting of transmembrane proteins (TMPs) on the trans-Golgi network and tubular sorting endosomes. The seven typical mammalian BDCPs share a predicted alpha-solenoid-beta propeller structure, suggesting they have a protocoatomer origin and function. We map the subcellular localization of seven BDCPs based on their dynamic colocalization with RAB and ARF small GTPases and identify five typical BDCPs on subdomains of dynamic tubular-vesicular compartments on the intersection of endocytic recycling and post-Golgi secretory pathways. We demonstrate that BDCPs interact directly with the cytosolic tails of selected TMPs and identify a subset of TMPs, whose trafficking to the plasma membrane is affected in cells lacking BDCP. We propose that the competitive binding of BDCPs and clathrin coat adaptors to the cytosolic tails of TMPs, followed by their clustering to distinct subdomains of secretory/recycling tubules function as a mechanism for sorting of TMPs in pleomorphic tubular-vesicular compartments that lack a clathrin coat.

## Teaser

In this manuscript, Pankiv et al. identify BEACH domain–containing proteins (BDCPs) as novel sorting adaptors for transmembrane proteins (TMPs) in post-Golgi secretory and recycling endocytic pathways. They demonstrate that BDCPs and clathrin coat adaptors localize to distinct subdomains of secretory/recycling tubules, leading to the sorting of TMPs into distinct transport carriers.

## Introduction

Around one-fourth of all human proteins contain one or several transmembrane domain(s) (Dobson et al., 2015; Fagerberg et al., 2010) and undergo complex sorting events during their trafficking from the place of synthesis at the endoplasmic reticulum (ER) to their final destination (Pfeffer and Rothman, 1987). In general, sorting of transmembrane proteins (TMPs) involves the binding of adaptor proteins to short linear motifs in their cytosolic tail, followed by clustering of cargo adaptors and membrane bending into vesicular or tubular carriers facilitated by recruitment of helper proteins (Arora and Damme, 2021). Several cargo adaptors and helper proteins form distinct coat complexes, including COPI and COPII, which mediate vesicular transport between ER and Golgi compartments, Retromer and Retreaver complexes that mediate tubular-mediated recycling from endocytic compartments, and different types of clathrin coats, promoting the formation of coated vesicles from the plasma membrane (PM), endocytic, and late secretory compartments (Dell’Angelica and Bonifacino, 2019; Paczkowski et al., 2015). Although the proteins constituting these various coat complexes have little sequence homology, they are all enriched in alpha-solenoid and beta-propeller structural domains and are suggested to have a common evolutionary origin, referred to as the protocoatomer hypothesis (Devos et al., 2004; Rout and Field, 2017).

Both the Golgi apparatus and early/sorting endosomes generate transport carriers for protein delivery to the PM. Secretory cargo is transported to the PM either in direct Golgi-to-PM carriers, positive for RAB6A (Fourriere et al., 2019; Grigoriev et al., 2007; Miserey-Lenkei et al., 2010), or indirectly via a RAB11-dependent mechanism (Chen et al., 1998; Khvotchev et al., 2003; Lock and Stow, 2005; Urbé et al., 1993). Recycling from endocytic compartments to the PM involves either RAB4-dependent fast or RAB11-dependent slow recycling compartments (Green et al., 1997; Sönnichsen et al., 2000; Ullrich et al., 1996; van der Sluijs et al., 1992). The Golgi and early/sorting endosomes also exchange material, best exemplified by the secretion and recycling of Mannose-6-phosphate receptors (Brown et al., 1986; Ghosh et al., 2003). Yet, our mechanistic insight into the highly dynamic nature and complexity of such sorting events and the nature of the carriers that leave or enter these sorting hubs remains largely unknown. Tubular-vesicular structures that are adjacent to and morphologically and functionally linked to exit sites of the Golgi and early/sorting endosomes have been described on the electron microscopy level as the trans-Golgi network (TGN) (Griffiths and Simons, 1986) and tubular sorting endosomes (TSE) (Peden et al., 2004)/tubular endosomal network (TEN) (Raub et al., 1990; Stoorvogel et al., 1996), respectively. Both, TGN and TSE/TEN have large surface-to-volume ratios and transform into transport carriers directed to spatially different acceptor compartments upon entrance of secretory or recycling cargo (Guo et al., 2014; Solinger and Spang, 2022; Stalder and Gershlick, 2020). While Retromer, Retriever, and related complexes have been suggested to mediate the sorting of transmembrane cargo into tubular carriers in early/sorting endosomes (Bonifacino and Hurley, 2008; Chen et al., 2019; McNally et al., 2017), no sorting machinery for tubular carriers at the TGN has been described so far. Such Golgi-derived tubular carriers, often referred to as pleomorphic tubular-vesicular carriers (Hirschberg et al., 1998; Stalder and Gershlick, 2020), have been established as the main transport compartment for delivery of secretory cargo from the Golgi to the PM (Hirschberg et al., 1998; Stalder and Gershlick, 2020), while TGN-derived clathrin-coated vesicular carriers are believed to transport proteins between the Golgi and endosomes (Kim and Gadila, 2016).

The complexity of the eukaryotic endomembrane system makes it difficult to differentiate the multitude of intracellular compartments based only on their morphology or subcellular localization. Ras-associated binding (RAB) and ADP-ribosylation factor (ARF) proteins are two large families of small GTPases that by shuttling between GTP- and GDP-bound states function as molecular switches that dynamically associate with the cytosolic surface of specific intracellular compartments and are commonly used to define the properties of those compartments (Stenmark, 2009; Sztul et al., 2019).

Beige and Chediak-Higashi syndrome (BEACH) domain-containing proteins (BDCPs) make up a family of large proteins characterized by the presence of a 300-amino acid (aa) large BEACH domain, identified in all branches of eukaryotes (Jogl et al., 2002; Nagle et al., 1996). The human protein family consists of seven typical members (LYST, ALFY/WDFY3, WDFY4, LRBA, NBEA, NBEAL1, and NBEAL2), one small (NSMAF/FAN), and one atypical (WDR81) member (Cullinane et al., 2013). The typical BDCPs are among the top 1% of all human proteins size-wise, ranging between 2.7 and 3.8 thousand aa. They all share the domain architecture of PH-BEACH-WD40 domains on the C-terminus and have a predicted concanavalin A (ConA)-like domain in the N-terminal or middle part of the protein (Burgess et al., 2009; Cullinane et al., 2013). Loss of function mutations in six out of the seven typical BDCPs cause early onset monogenic diseases and are strongly removed from the human gene pool (Kahr et al., 2011; Karczewski et al., 2020; Le Duc et al., 2019; Lopez-Herrera et al., 2012; Mulhern et al., 2018; Nagle et al., 1996; Theisen et al., 2018), while SNPs in the seventh member, NBEAL1, are associated with increased risk of coronary artery disease (Bindesbøll et al., 2020). Although different BDCPs have been associated with regulation of vesicles fusion and fission (Cullinane et al., 2013), autophagy (Simonsen et al., 2004), antigen cross-presentation (Theisen et al., 2018), regulation of PI3K activity (Liu et al., 2016) or signaling from transmembrane receptors (Adam-Klages et al., 1996), their subcellular localization and molecular functions are poorly characterized due to their large size. The similar structural organization of BDCPs may suggest that a common functional mechanism underlies the diverse phenotypic manifestation of loss of function for different BDCPs. The BDCP LRBA was recently identified as the regulator of CTLA4 immune checkpoint trafficking and was suggested to compete with AP1 clathrin coat adaptors for binding to the cytosolic tail of CTLA4 (Lo et al., 2015). Also, other BDCPs have been shown to directly bind to TMPs (Adam-Klages et al., 1996; Bindesbøll et al., 2020; del Pino et al., 2011) or regulate their trafficking (Delage et al., 2023; Gromova et al., 2018; Lo et al., 2018; Nair et al., 2013), indicating their involvement in intracellular membrane trafficking.

In the current study, we characterized typical BDCPs as novel TMPs cargo adaptors of pleomorphic tubular-vesicular carriers originating from the TGN and TSE/TEN.

## Results

### Localization of human BEACH domain-containing proteins

**Figure 1.**
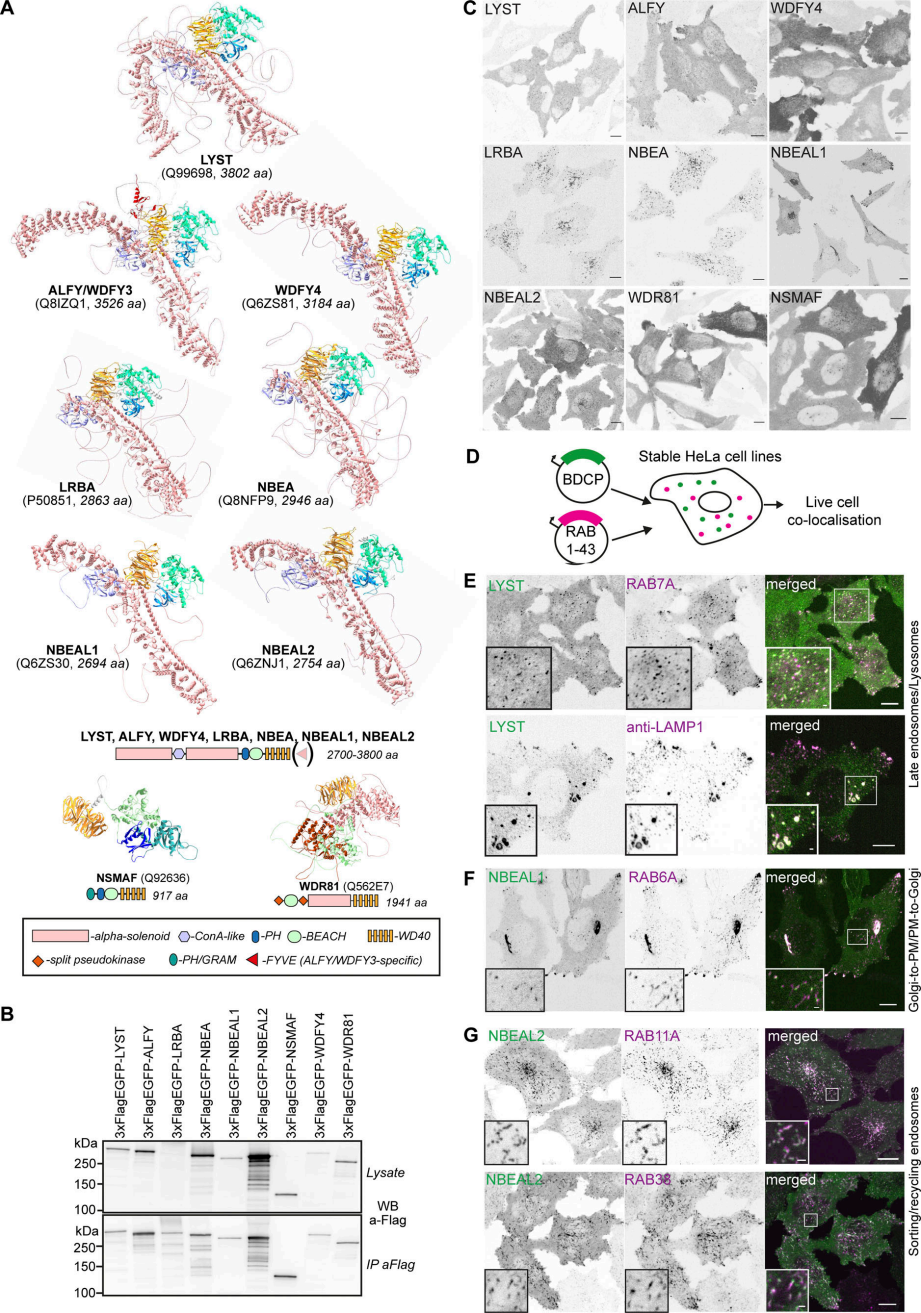
**Mapping the cellular localization of BDCPs, a family of large cytosolic alpha-solenoid/beta-propeller domain proteins. (A)** Alpha-fold models and schematic structures of typical, atypical, and small BDCPs. **(B)** Western blot and anti-flag immunoprecipitation of cell lysates from HeLa cells stably expressing 3xFlag-EGFP-tagged full-length human BDCPs. **(C)** Representative confocal images of HeLa cells stably expressing 3xFlag-EGFP-tagged human BDCPs. Scale bars 10 µm. **(D)** Schematic of the live-cell imaging screen done to characterize the nature of BDCP-positive compartments. HeLa cells stably expressing a single BDCP and one of 42 RAB small GTPase fused to 3xFlag-EGFP and mScarlet, respectively, were imaged live using a spinning-disc confocal microscope to detect colocalization and co-migration of both proteins. See Table 1 for an overview of the screen results. **(E)** HeLa cells stably expressing tdNG-LYST and mScarlet-RAB7A were imaged live (upper panel) or fixed and stained with an anti-LAMP1 antibody (lower panel). Scale bars 10 µm (main figure) or 1 µm (inset magnifications). **(F)** HeLa cells stably expressing 3xFlag-EGFP-NBEAL1 and mScarlet-RAB6A were imaged live. Scale bars 10 µm (main figure) or 1 µm (inset magnifications). **(G)** HeLa cells stably expressing 3xFlag-EGFP-NBEAL2 and mScarlet-RAB11A (upper panel) or -RAB38 (lower panel) were imaged live. Scale bars 10 µm (main figure) or 1 µm (inset magnifications). Source data are available for this figure: SourceData F1.

The localization and function of the nine human BEACH domain-containing proteins (BDCPs) remain largely unknown as their large size makes cloning and expression of full-length proteins difficult and due to the lack of specific antibodies for staining of endogenous proteins. Using the AlphaFold protein structure prediction (Jumper et al., 2021), we found that the seven largest BDCPs (LYST, ALFY, WDFY4, LRBA, NBEA, NBEAL1, NBEAL2, referred to as typical BDCPs) share a similar structural organization, despite having generally <15% sequence identity beyond the BEACH domain. Their N-terminus and middle part are dominated by a large extended alpha-solenoid and a ConA-like domain, followed by a PH-BEACH domain assembly and WD40 repeats at the C-terminus (Fig. 1 A). The smallest BDCP (NSMAF) has an N-terminal PH/GRAM-like domain, followed by PH-BEACH-WD40-domains but lacks alpha-solenoid and ConA-like domains (Fig. 1 A). The domain organization of the atypical BDCP WDR81 resembles that of VPS15/PIK3R4, with an N-terminal pseudokinase domain, followed by an alpha solenoid and WD40 repeats, but unlike VPS15/PIK3R4, WDR81 has a BEACH domain inserted in the activation loop of the pseudokinase (Fig. 1 A).

Proteins having a similar structure often have a common function. To identify the cellular localization of the individual BDCPs and their possible function, we cloned all nine BDCPs in frame with an N-terminal 3xFlag-EGFP tandem tag, except for LYST, which was tagged either to an N-terminal 3xFlag-EGFP or to a tandem dimer of NeonGreen (tdNG), and WDR81, which was cloned with a C-terminal EGFP-3xFlag tag, and generated stably transfected HeLa cells (Fig. 1 B). While LYST, ALFY, LRBA, NBEA, NBEAL1, NBEAL2, and NSMAF all displayed distinct localizations to tubular-vesicular cytoplasmic structures as analyzed by live cell imaging, WDFY4 and WDR81 showed diffuse cytosolic staining (Fig. 1 C).

To further assess the nature and functional properties of the tubular-vesicular structures marked by the individual BDCPs, we cloned 42 RAB small GTPases, well-known markers of different endocytic and secretory compartments (Stenmark, 2009) as mScarlet-I fusion proteins and generated a series of HeLa cells stably expressing combinations of a single mScarlet-I-RAB protein together with one of the seven 3xFlag-EGFP tagged BDCPs (Fig. 1 D). Using live-cell imaging of the double-transfected cells, we localized the different BDCPs to specific cellular compartments as summarized in Table 1.

**Table 1.** Colocalization of BDCPs with organelle marker proteins. nt: not tested, −: no colocalization, +: colocalization, +/−: partial colocalization

	LYST	ALFY	LRBA	NBEA	NBEAL1	NBEAL2	NSMAF
RAB1A	−	−	−	−	+/− Golgi	−	−
RAB2A	nt	−	−	−	+/− Golgi	−	−
RAB3A	−	−	−	−	+/− Golgi	−	−
RAB4A	−	−	+/− Cytosolic tubules	+/− Cytosolic tubules	−	−	+/− Periniclear vesicles
RAB5A	−	+/− Cytosolic vesicles	−	−	−	−	+
RAB6A	−	+/− Vesicles enriched in cell protrusion	+/− Golgi-connected tubules	+/− Golgi- connected tubules	+	−	−
RAB7A	+/− Cytosolic vesicles	−	−	−	−	−	−
RAB8A	−	+/− Cytosolic tubules	−	−	−	−	−
RAB9A	nt	−	−	−	−	−	−
RAB10	nt	−	−	−	−	−	−
RAB11A	−	−	+/− Cytosolic tubules	+/− Cytosolic tubules	−	+	−
RAB12	nt	−	−	−	+/− Golgi	−	−
RAB13	nt	−	−	−	−	−	−
RAB14	nt	nt	+/− Cytosolic tubules	+/− Cytosolic tubules	−	−	+
RAB15	nt	−	−	−	+/− Golgi	−	−
RAB17	nt	−	nt	nt	−	nt	nt
RAB18	nt	−	−	−	−	−	−
RAB19	nt	−	−	−	−	+ Cytosolic vesicles	−
RAB20	nt	−	−	−	+/− Golgi	−	−
RAB21	nt	+/− Cytosolic vesicles	nt	nt	−	nt	nt
RAB22A	nt	+/− Cytosolic vesicles	−	−	−	−	+/− Perinuclear vesicles
RAB23	nt	−	−	−	−	−	−
RAB24	nt	−	−	−	−	−	−
RAB25	nt	−	+/− Cytosolic tubules	+/− Cytosolic tubules	−	+	−
RAB26	nt	−	nt	−	−	−	−
RAB27A	nt	−	−	−	−	+ Cytosolic vesicles	−
RAB28	nt	−	−	−	−	−	−
RAB29	nt	−	−	−	+/− Golgi	−	−
RAB30	nt	−	−	−	+/− Golgi	−	−
RAB31	nt	−	−	−	+/− Golgi	−	−
RAB32	nt	−	−	−	+/− Golgi	−	−
RAB33B	nt	nt	−	−	+/− Golgi	−	−
RAB34	nt	−	nt	−	−	−	−
RAB35	nt	+/− Cytosolic tubules	−	−	−	−	−
RAB36	nt	−	nt	−	−	−	−
RAB37	nt	−	nt	nt	+/− Golgi	nt	nt
RAB38	nt	−	nt	−	+/− Golgi	+	−
RAB39A	nt	−	nt	nt	−	nt	nt
RAB40A	nt	nt	nt	−	−	nt	−
Rab41	nt	−	nt	nt	−	nt	nt
RAB42	nt	nt	nt	nt	−	nt	nt
RAB43	nt	+/− Vesicles	−	−	+/− Golgi	−	−
ARF1	nt	nt	+	+	+/− Golgi and Golgi-connected tubules	−	nt
ARF3	nt	nt	+/− Cytosolic tubules	nt	+/− Golgi and Golgi-connected tubules	nt	nt
ARF4	nt	nt	−	nt	+/− Golgi	nt	nt
ARF5	nt	nt	−	nt	+/− Golgi	nt	nt
ARF6	nt	nt	−	nt	−	nt	nt
AP1M1	nt	nt	+ Beads on a string	+ Beads on a string	+/− Beads on a string on Golgi-connected tubules	−	nt
AP2M1	nt	nt	−	nt	−	nt	nt
AP3M1	nt	nt	+/− Beads on a sting	+/− Beads on a string	+/− Beads on a string on Golgi-connected tubules	nt	nt
AP4M1	nt	nt	−	nt	−	nt	nt
GGA1	nt	nt	+/− Beads on a sting	+/− Beads on a string	+/− Beads on a string on Golgi-connected tubules	nt	nt
GGA2	nt	nt	−	−	−	nt	nt
GGA3	nt	nt	+/− Beads on a string	+/− Beads on a string	+/− Beads on a string on Golgi-connected tubules	nt	nt
EpsinR	nt	nt	+/− Beads on a string	+/− Beads on a string	+/− Beads on a string on Golgi-connected tubules	nt	nt
Clathrin heavy chain	nt	nt	+/− Beads on a string	+/− Beads on a string	+/− Beads on a string on Golgi-connected tubules	nt	nt

The specific compartment(s) where colocalization occurs is indicated.

**Figure S1.**
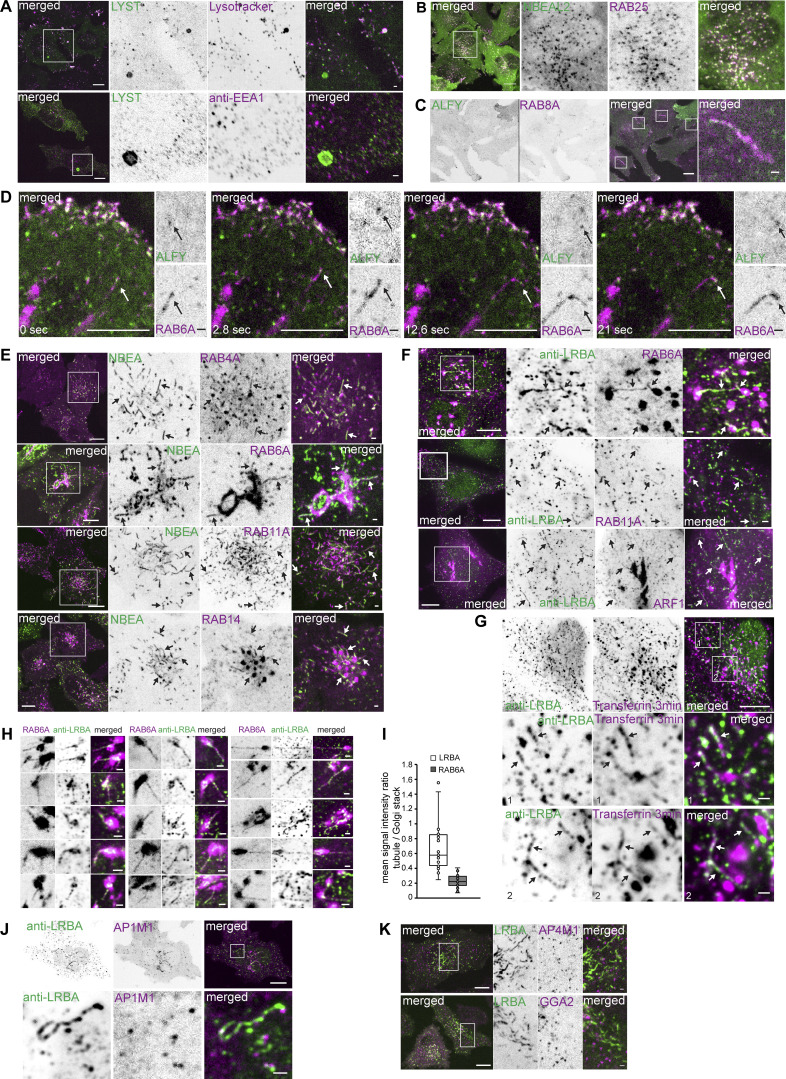
**Mapping the cellular localization of BDCPs. (A)** HeLa cells stably transfected with LYST tagged with a tandem dimer NeonGreen were treated for 30 min with 20 nM of Lysotracker Deep Red and imaged live (upper panel) or fixed and stained with an anti-EEA1 antibody (lower panel). Scale bar 10 µm (left merged) or 1 µm (right merged). **(B)** HeLa cells stably transfected with 3xFlag-EGFP-NBEAL2 and mScarlet-I-RAB25 were imaged live. Scale bar 10 µm (left merged) or 1 µm (right merged). **(C)** HeLa cells stably transfected with 3xFlag-EGFP-ALFY and mScarlet-I-RAB8A were imaged live. Scale bar 10 µm (left merged) or 1 µm (right merged). **(D)** HeLa cells stably transfected with 3xFlag-EGFP-ALFY and mScarlet-RAB6A were imaged live. ALFY speckle co-migrates with the tip of RAB6A-positive tubule (arrows). Scale bar 10 µm (main figure) or 1 µm (inset magnifications). **(E)** HeLa cells stably transfected with 3xFlag-EGFP-NBEA and mScarlet tagged RAB4A, RAB6A, RAB11A, or RAB14 were imaged live. NBEA partially colocalizes with the tubular segments of RAB4A-, RAB11A,- or RAB14- positive endosomes and RAB6A-positive Golgi-connected tubules (arrows). Scale bar 10 µm (left merged) or 1 µm (right merged). **(F)** HeLa cells stably transfected with the indicated small GTPases were fixed and stained with an anti-LRBA antibody. Endogenous LRBA colocalizes with mScarlet-RAB6A, mScarlet-RAB11A and ARF1-mScarlet on cytosolic tubules (arrows). Scale bar 10 µm (left merged) or 1 µm (right merged). **(G)** HeLa cells fixed 3 min after the addition of 10 µg/ml of AlexaFluor647-Transferrin to the culture media and stained with an anti-LRBA antibody. Scale bar 10 µm (upper merged) or 1 µm (middle and lower merged. **(H)** Examples of mScarlet-RAB6A-positive Golgi and Golgi-connected tubules that colocalize with endogenous LRBA as in F upper panel. Scale bars 1 µm. **(I)** Quantification of the mean signal intensity ratio of LRBA or RAB6A signals on LRBA/RAB6A positive tubule vs. nearby LRBA/RAB6A positive Golgi stack. The mean signal intensity of green (endogenous LRBA) or magenta (mScarlet-RAB6A) channels within a 4 × 4 pixels rectangular box over the tubule or Golgi stack was used for calculations. *n* = 20. **(J)** HeLa cells stably transfected with AP1M1-mScarlet stained with an antibody against endogenous LRBA. Scale bar 10 µm (upper merged) or 1 µm (lower merged). **(K)** HeLa cells stably transfected with 3xFlag-EGFP-LRBA and AP4M1-mScarlet or GGA2-mScarlet imaged live. Scale bars 10 µm (left merged) or 1 µm (right merged).

tdNG-LYST localized to several small and large cytosolic structures that stained positive for mScarlet-RAB7A and endogenous LAMP1 (Fig. 1 E), as well as Lysotracker Deep Red (Fig. S1 A, upper panel), but not for EEA1 (Fig. S1 A, lower panel). The identification of LYST-positive structures as late endosomes and/or lysosomes is not surprising as cells lacking LYST are characterized by enlarged lysosomes (Holland et al., 2014; Serra-Vinardell et al., 2023; White, 1966).

In line with our previous identification of NBEAL1 as a Golgi localized protein (Bindesbøll et al., 2020), we found a strong colocalization between NBEAL1 and Golgi-specific RAB GTPases (Table 1). RAB6A was the only RAB that also colocalized with NBEAL1 on Golgi-connected tubules and Golgi-derived vesicles that could be tracked to and accumulated at cell protrusions (Fig. 1, C and F; and Video 1). Additionally, a fraction of NBEAL1- and RAB6A-positive vesicles were observed moving in a retrograde PM-to-Golgi direction (Video 1), suggesting that NBEAL1 positive structures are identical to RAB6-containing secretory Golgi-to-PM carriers (Fourriere et al., 2019; Grigoriev et al., 2007; Miserey-Lenkei et al., 2010) and recycling PM-to-Golgi retrograde vesicles (Carpier et al., 2018; Huber et al., 2020; Mallard et al., 2002).

**Video 1.** **NBEAL1 (green) and RAB6A (magenta) colocalize on Golgi-to-PM anterograde (arrows left cell) and PM-to-Golgi retrograde (arrows right cell) carriers.** HeLa cells stably transfected with 3xFlag-EGFP-NBEAL1 and mScarlet-RAB6A were imaged live. Scale bar 10 µm. Playback speed 7x.

NBEAL2 displayed strong colocalization with two members of the RAB11-family (RAB11A and RAB25) that are commonly described as markers of recycling endosomes (Kelly et al., 2012; Ullrich et al., 1996), as well as with RAB38, a marker of transport carriers to lysosome-related organelles (LRO) (Bultema et al., 2012; Bultema and Di Pietro, 2013; Wasmeier et al., 2006) (Fig. 1 G and Fig. S1 B; and Table 1). Interestingly, we observed no colocalization of NBEAL2 with the closely related RAB32 (Table 1).

**Figure 2.**
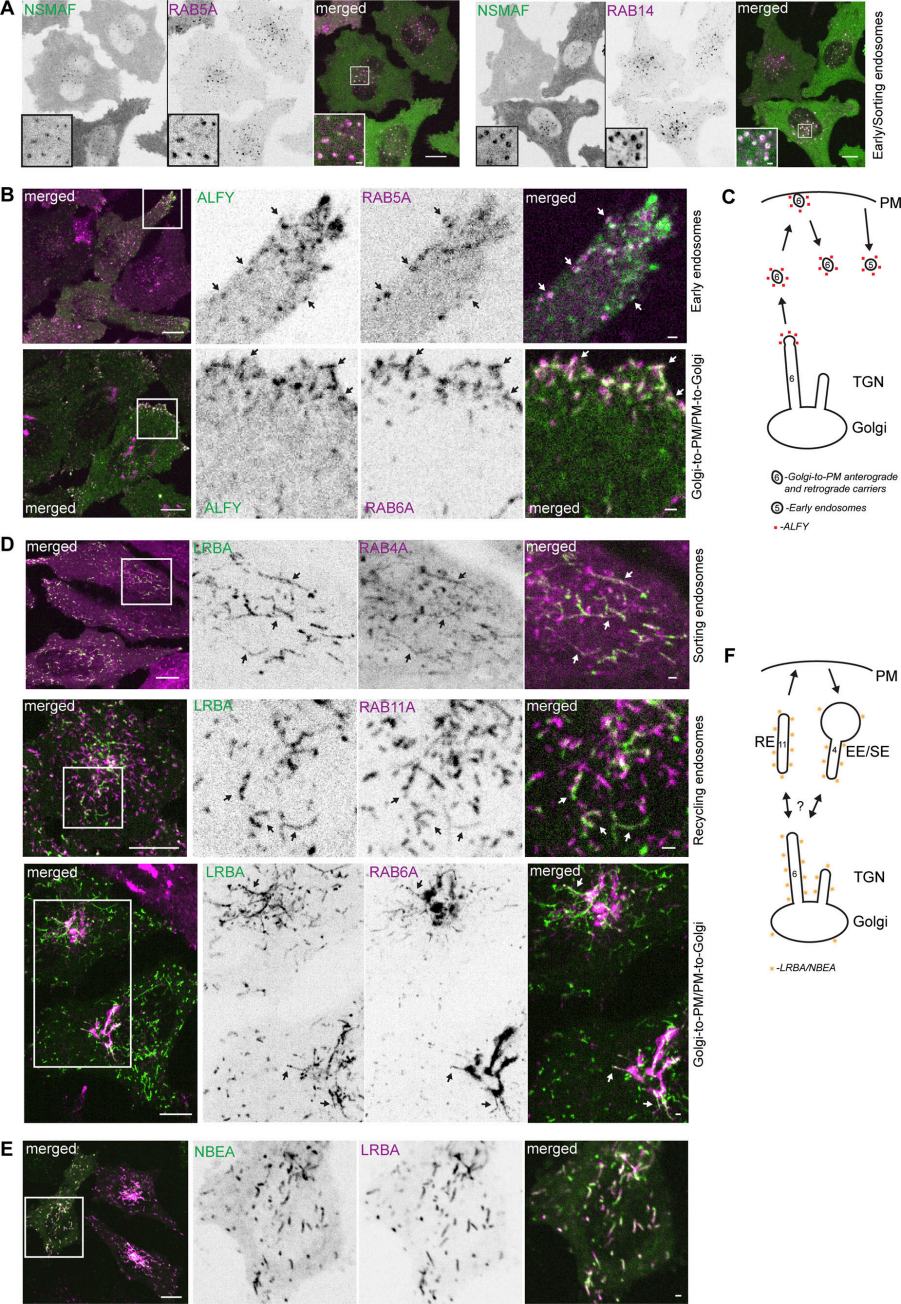
**BDCPs localize to both secretory and endocytic/recycling compartments. (A)** HeLa cells stably expressing 3xFlag-EGFP-NSMAF and mScarlet-RAB5A (left panel) or -RAB14 (right panel) were imaged live. Scale bars 10 µm (main figure) or 1 µm (inset magnifications). **(B)** HeLa cells stably expressing 3xFlag-EGFP-ALFY and mScarlet-RAB5A (upper panel) or -RAB6A (lower panel) were imaged live. Scale bars 10 µm (left merged) or 1 µm (right merged). **(C)** Schematics showing localization of ALFY with RAB5 and RAB6. PM- plasma membrane, TGN- trans-Golgi network. **(D)** HeLa cells stably expressing 3xFlag-EGFP-LRBA and mScarlet-RAB4A, -RAB11A, or -RAB6A were imaged live. Scale bars 10 µm (left merged) or 1 µm (right merged). **(E)** HeLa cells stably expressing 3xFlag-EGFP-NBEA and mScarlet-LRBA were imaged live. Scale bar 10 µm (left merged) or 1 µm (right merged). **(F)** Schematics showing localization of LRBA and NBEA with RAB4, RAB11 and RAB6. PM- plasma membrane, EE/SE- early/sorting endosomes, RE- recycling endosomes, TGN- trans-Golgi network.

NSMAF was originally identified as a protein associated with the cytosolic tail of the TNF receptor on the plasma membrane (Adam-Klages et al., 1996). We failed to see the typical plasma membrane staining of NSMAF, but did detect its strong colocalization with endosomal RAB5A and RAB14, as well as a partial colocalization with RAB4A and RAB22 on perinuclear vesicles (Fig. 2 A and Table 1), indicating that NSMAF is an early/sorting endosomal protein.

We recently reported a partial colocalization of ALFY with RAB5A (Søreng et al., 2022), which we confirmed in this study (Fig. 2 B upper panel and Video 2). Furthermore, we observed a partial colocalization of ALFY with RAB22, another marker of early endosomes, and with RAB6A on highly dynamic vesiculo-tubular structures in cell protrusions close to the plasma membrane (Fig. 2 B lower panel, Video 3 and Table 1), as well as with RAB8A-positive tubular structures (Fig. S1 C and Table 1). Unlike NBEAL1 (Fig. 1 F), ALFY did not colocalize with RAB6A-positive Golgi or Golgi-linked tubules but could occasionally be detected on the tip of growing RAB6A-positive tubules (Fig. S1 D). Colocalization of ALFY with markers of peripheral Golgi-to-plasma membrane secretory carriers and early endosomes suggests that it may function at the intersection of the late secretory and early endocytic pathways (Fig. 2 C).

**Video 2.** **ALFY (green) and RAB5A (magenta) partially colocalize on cytosolic vesicles.** HeLa cells stably transfected with 3xFlag-EGFP-ALFY and mScarlet-RAB5A were imaged live. Scale bar 1 µm. Playback speed 21x.

**Video 3.** **ALFY (green) and RAB6A (magenta) partially colocalize in cell protrusions on highly dynamic tubulo-vesicular structures.** HeLa cells stably transfected with 3xFlag-EGFP-ALFY and mScarlet-RAB6A were imaged live. Scale bar 1 µm. Playback speed 21x.

LRBA and NBEA demonstrated a highly similar pattern of partial colocalization with three sets of RABs, including the tubular component of the sorting endosomal markers RAB4A and RAB14, a partial colocalization with the recycling endosomal markers RAB11 and RAB25, and with Golgi-linked tubules positive for RAB6A (Fig. 2 D and Fig. S1 E; and Table 1). Importantly, endogenous LRBA showed a similar colocalization pattern (Fig. S1 F). We could also observe a weak colocalization of LRBA or NBEA with RAB6A-positive Golgi cisterns (Fig. 2 D; and Fig. S1, F, H, and I). Live cell imaging of HeLa cells stably expressing both EGFP-LRBA and mScarlet-NBEA confirmed that these proteins localize to the same compartments (Fig. 2, E and F), indicating they may have redundant functions.

### Clathrin-coated vesicles bud from BDCP-positive tubules

Since several of the typical BDCPs localize to Golgi and Golgi-linked tubular-vesicular structures, we further characterized those BDCP-positive compartments for colocalization with ARF small GTPases and markers of COPI, COPII, and clathrin-coated vesicles (CCVs) that are known to regulate protein and membrane trafficking to and from the Golgi (Paczkowski et al., 2015; Wong-Dilworth et al., 2023). The respective proteins were tagged with mScarlet and co-expressed in HeLa cells stably expressing single 3xFlag-EGFP-tagged BDCPs, and their colocalization was analyzed by live-cell imaging (Table 1).

**Figure 3.**
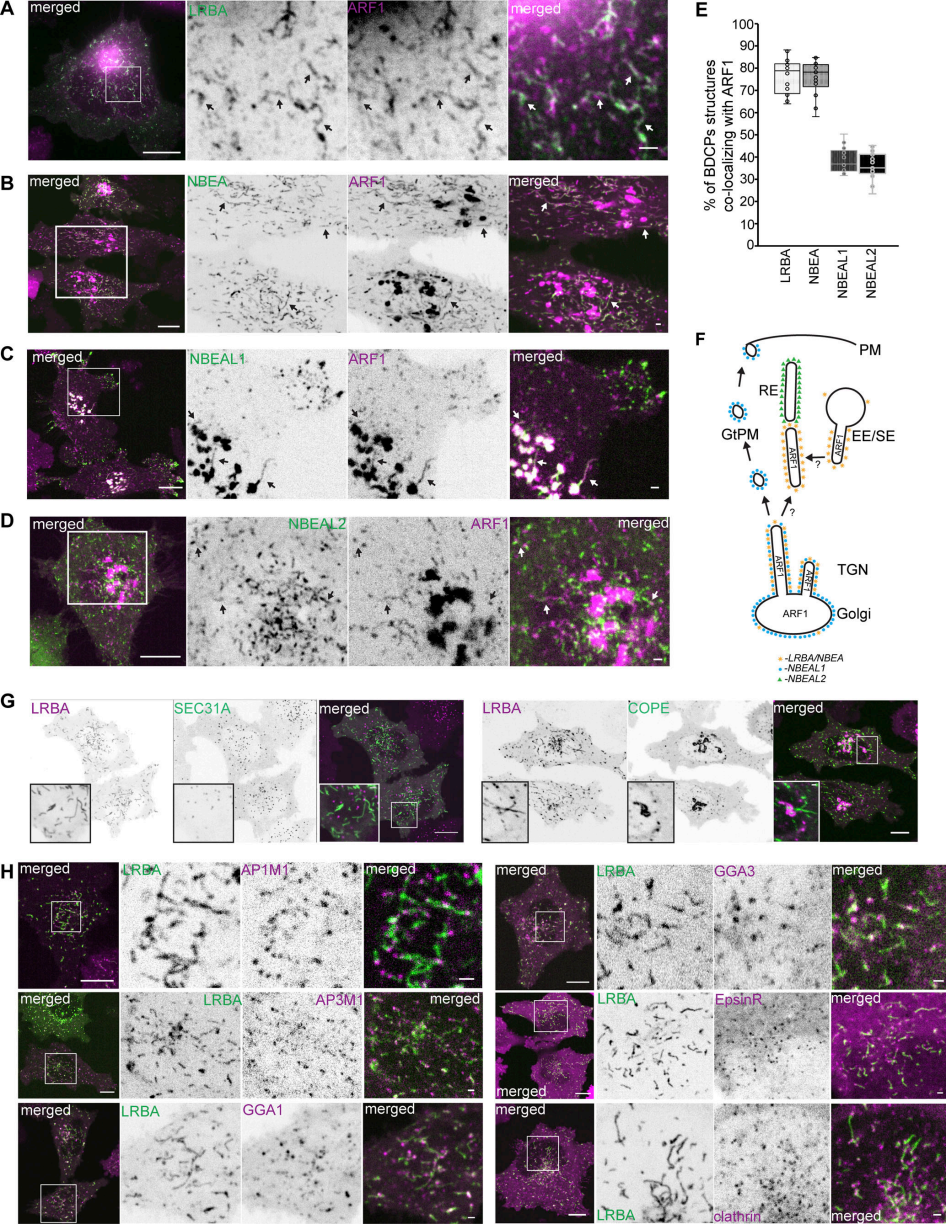
**LRBA-positive tubules are highly dynamic sites of clathrin-coated vesicle formation. (A)** HeLa cells stably expressing 3xFlag-EGFP-LRBA and ARF1-mScarlet were imaged live. Scale bars 10 µm (left merged) or 1 µm (right merged). **(B)** HeLa cells stably expressing 3xFlag-EGFP-NBEA and ARF1-mScarlet were imaged live. Scale bars 10 µm (left merged) or 1 µm (right merged). **(C)** HeLa cells stably expressing 3xFlag-EGFP-NBEAL1 and ARF1-mScarlet were imaged live. Scale bars 10 µm (left merged) or 1 µm (right merged). **(D)** HeLa cells stably expressing 3xFlag-EGFP-NBEAL2 and ARF1-mScarlet were imaged live. Scale bars 10 µm (left merged) or 1 µm (right merged). **(E)** Quantification of colocalization between structures positive for either LRBA, NBEA, NBEAL1 or NBEAL2, and ARF1. HeLa cells stably expressing ARF1-mScarlet and the indicated BDCP tagged with 3xFlag-EGFP were imaged with confocal microscopy. EGFP or mScarlet positive structures were segmented and each dot represent the percentage of BDCP positive objects in each cell partially or completely overlapping with ARF1 positive object. *n* ≥ 15 per condition. **(F)** Schematic summary of the colocalization of LRBA/NBEA, NBEAL1, and NBEAL2-positive tubular compartments with the clathrin/COPI coat recruitment factor ARF1. PM- plasma membrane, EE/SE- early/sorting endosomes, RE- recycling endosomes, GtPM- Golgi to plasma membrane carriers, TGN- trans-Golgi network. **(G)** HeLa cells stably expressing 3xFlag-EGFP-LRBA and mScarlet-SEC31A (COPII marker, left panel) or mScarlet-COPE (COPI marker, right panel) were imaged live. Scale bars 10 µm (main figure) or 1 µm (inset magnifications). **(H)** HeLa cells stably expressing 3xFlag-EGFP-LRBA together with clathrin coat adaptors AP1M1-mScarlet (upper left), AP3M1-mScarlet (middle left), GGA1-mScarlet (lower left), GGA3-mScarlet (upper right), mScarlet-EpsinR (middle right), and mScarlet-clathrin heavy chain (lower right) were imaged live. Scale bars 10 µm (left merged) or 1 µm (right merged).

Surprisingly, all LRBA-positive structures colocalized with ARF1 and partially with ARF3, but did not show any overlap with ARF4, ARF5, ARF6, or SAR1A (Fig. 3, A, E, and F and Table 1). Similarly, endogenous LRBA colocalized with ARF1-mScarlet-positive tubules attached to the Golgi or localized at the cell periphery (Fig. S1 F). We also observed complete colocalization of NBEA-positive structures with ARF1 (Fig. 3, B and E). In contrast, NBEAL1 colocalized with ARF1 only on Golgi and Golgi-linked tubules, but not on peripheral vesicles (Fig. 3, C and E) and did not colocalize with other ARFs or SAR1A (Table 1). NBEAL2 and ARF1 localized to distinct, sometimes closely adjacent structures, resembling different domains of the same tubule with little signal overlap (Fig. 3 D).

**Figure 4.**
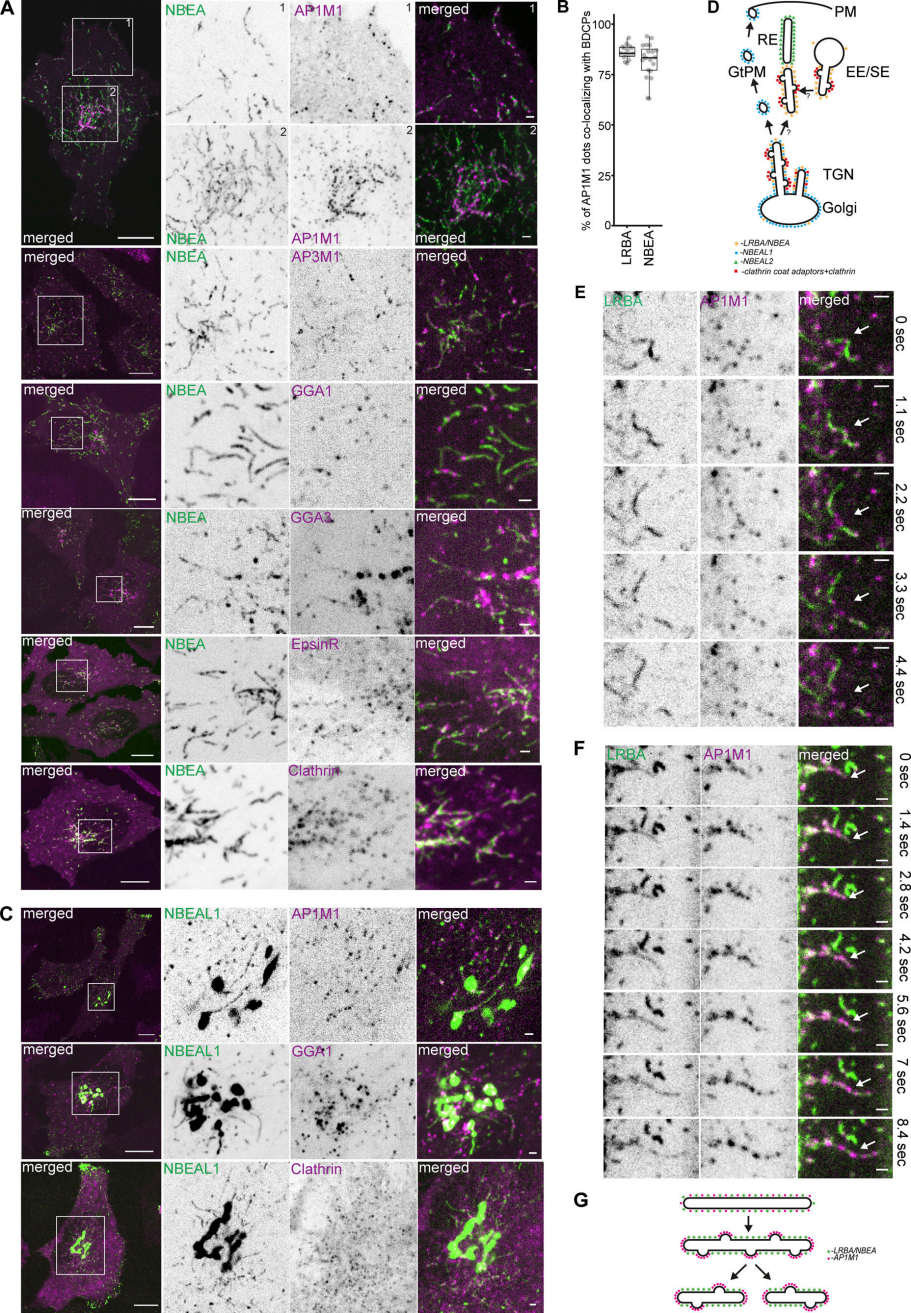
**NBEA-positive tubules are highly dynamic sites of clathrin-coated vesicle formation. (A)** HeLa cells stably expressing 3xFlag-EGFP-NBEA together with clathrin coat adaptors AP1M1-mScarlet (1), AP3M1-mScarlet (2), GGA1-mScarlet (3), GGA3-mScarlet (4), mScarlet-EpsinR (5), and mScarlet-clathrin heavy chain (6) were imaged live. Scale bar 10 µm (left merged) or 1 µm (right merged). **(B)** Quantification of the percentage of AP1M1 dots colocalizing with BDCPs in HeLa cells expressing 3xFlag-EGFP-LRBA or 3xFlag-EGFP-NBEA together with AP1M1-mScarlet (data shown in Fig. 3 H or Fig. 4 A, respectively). *n* = 15–20 cells and 150–300 AP1M1 dots/cell were quantified. **(C)** HeLa cells stably expressing 3xFlag-EGFP-NBEAL1 and mScarlet-tagged AP1M1 (1), GGA1 (2), or clathrin heavy chain (3) were imaged live. Scale bars 10 µm (left merged) or 1 µm (right merged). **(D)** Schematic summary of the localization of LRBA/NBEA, NBEAL1, and NBEAL2-positive tubular compartments with clathrin coat adaptors and clathrin. PM- plasma membrane, EE/SE- early/sorting endosomes, RE- recycling endosomes, GtPM- Golgi to plasma membrane carriers, TGN- trans-Golgi network. **(E)** LRBA- and AP1M1-positive tubules undergo rapid fission at or close to AP1M1-positive speckles (indicated by arrows). HeLa cells stably transfected with 3xFlag-EGFP-LRBA and mScarlet-AP1M1 were imaged live with representative snap-shot images shown. Scale bars 1 µm. **(F)** LRBA and AP1M1 partition in a mutually exclusive manner along the length of growing LRBA and AP1M1 positive tubule. HeLa cells stably transfected with 3xFlag-EGFP-LRBA and mScarlet-AP1M1 were imaged live with representative snap-shot images shown. Scale bars 1 µm. **(G)** Schematic summary of the dynamics of LRBA and AP1M1 positive tubule from Fig. 4, E and F.

**Figure S2.**
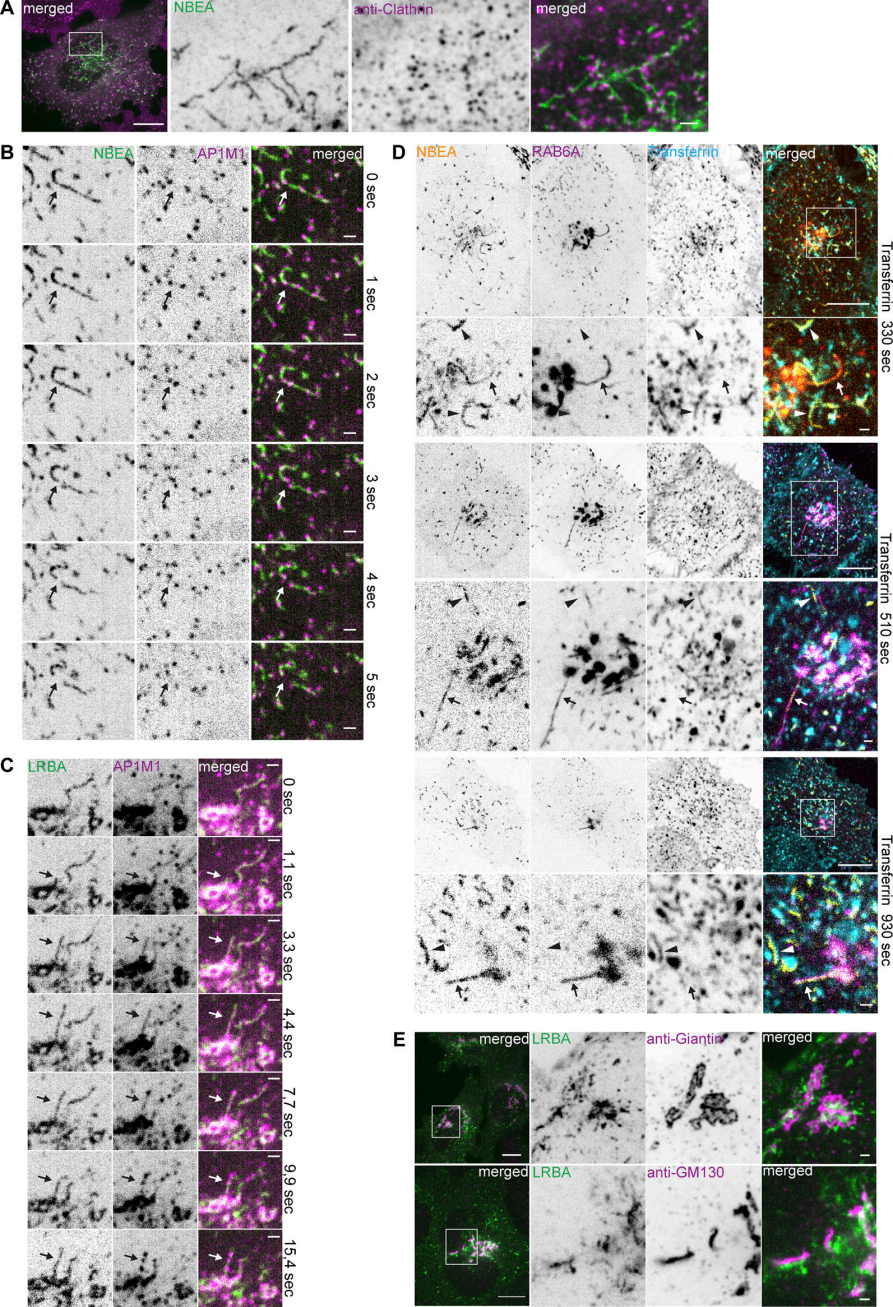
**Characterization of LRBA- and NBEA- positive compartments. (A)** HeLa cells stably transfected with 3xFlag-EGFP-NBEA were fixed and stained with an anti-clathrin antibody. Scale bar 10 µm (left merged) or 1 µm (right merged). **(B)** HeLa cells stably transfected with 3xFlag-EGFP-NBEA and AP1M1-mScarlet were imaged live with 1 s intervals. NBEA- and AP1M1-positive tubules undergo rapid fission at or close to AP1M1-positive speckles (arrow). Scale bars 1 µm. **(C)** Live cell imaging of HeLa cells stably transfected with 3xFlag-EGFP-LRBA and AP1M1-mScarlet. LRBA and AP1M1 into distinct subdomains along the length of the growing tubule (arrows). Scale bars 1 µm. **(D)** HeLa cells stably transfected with 3xFlag-EGFP-NBEA and mScarlet-RAB6A were imaged live with 1 min intervals starting 30 s after supplementation of growth media with 10 µg/ml of AlexaFluor647-Transferrin. NBEA-decorated tubules, positive for RAB6A (arrow) do not accumulate transferrin (arrowhead). Scale bars 10 µm (main figures) or 1 µm (zoomed in inserts). **(E)** HeLa cells stably transfected with 3xFlag-EGFP-LRBA were fixed and stained with anti-Giantin (upper panel) or anti-GF130 (lower panel) antibodies. Endogenous markers of media-Golgi (giantin) and cis-Golgi (GM130) localize in close proximity to perinuclear 3xFlag-EGFP-LRBA positive structures but do not colocalize with LRBA-positive tubules. Scale bars 10 µm (left merged) or 1 µm (right merged).

ARF1 is known to mediate the recruitment of both COPI and clathrin coats to membranes (Ren et al., 2013; Yu et al., 2012). Neither LRBA nor NBEAL1 colocalized with markers of COPI- and COPII-coated vesicles (COPE or SEC31A, respectively) (Fig. 3 G and Table 1). In contrast, over 80% of the clathrin coat adaptor AP1M1 positive speckles localized along or to the ends of tubules decorated with LRBA, both in stably transfected cells (Fig. 3 H and Fig. 4 B and Video 4) and when staining for endogenous proteins (Fig. S1 J). We also observed partial colocalization of LRBA with other clathrin coat adaptors, including AP3M1, GGA1, GGA3, and EpsinR, as well as clathrin heavy chain, in a beads-on-a-string fashion, but not with AP2M1, AP4M1 or GGA2 (Fig. 3 H and Fig. S1 K; and Table 1). Intriguingly, similar to LRBA, NBEA also showed a strong beads-on-a-string colocalization pattern with AP1M1 both in the perinuclear region and the cell periphery (Fig. 4 A) with partial colocalization with AP3M1, GGA1, GGA3, EpsinR, and clathrin heavy chain (Fig. 4 A and Fig. S2 A), and no colocalization with AP2M1, AP4M1, or GGA2 (Table 1). Golgi-connected NBEAL1 positive tubules, but not peripherally localized vesicles, also displayed a partial colocalization with AP1M1, AP3M1, GGA1, GGA3, and EpsinR in a beads-on-a-string fashion (Fig. 4, C and D; and Table 1), in line with colocalization of NBEAL1-positive tubules, but not vesicles, with ARF1. We failed to detect any colocalization of NBEAL2 positive compartments with AP1M1 (Fig. 4 D and Table 1).

**Video 4.** **NBEA- and AP1M1-positive structures can accumulate endocytosed transferrin.** HeLa cells stably transfected with 3xFlag-EGFP-NBEA and AP1M1-mScarlet were imaged live 20 min after addition of 10 µg/ml of AlexaFluor647-Transferrin to the media. Scale bar 10 µm. Playback speed 17x.

The strong colocalization of AP1M1 with LRBA/NBEA positive tubules in beads-on-a-string manner indicates that the majority of AP1M1-positive speckles represent clathrin-coated buds attached to the donor membrane and not separate CCV (Fig. 4 D). Using live cell imaging, we observed that most of the LRBA/NBEA and AP1M1 positive tubules were highly dynamic and underwent multiple fission and what looked like fusion events (Fig. 4 E and Fig. S2 B and Video 4), although the latter is difficult to confirm due to the high mobility and dynamic shape of the observed structures. Interestingly, the observed fission events occurred at or close to AP1M1 positive buds along the tubule, resulting in two smaller tubules, each containing AP1M1 signal on the newly formed ends (Fig. 4, E and G; and Fig. S2 B). We also observed the formation and growth of new LRBA and AP1M1 positive tubules from the perinuclear LRBA/AP1M1 positive mass (Fig. 4 F and Fig. S2 C). Limited by the resolution of light microscopy, we observed an initial diffuse distribution of AP1M1 and LRBA along the growing tubule, followed by sequestration of both proteins in distinct subdomains along the length of the tubule (Fig. 4, F and G; and Fig. S2 C). Taken together, our data indicate that LRBA, NBEA, and NBEAL1 positive tubules are major sites of CCV formation.

### LRBA and NBEA positive compartments receive input from both endocytic and secretory pathways

**Figure 5.**
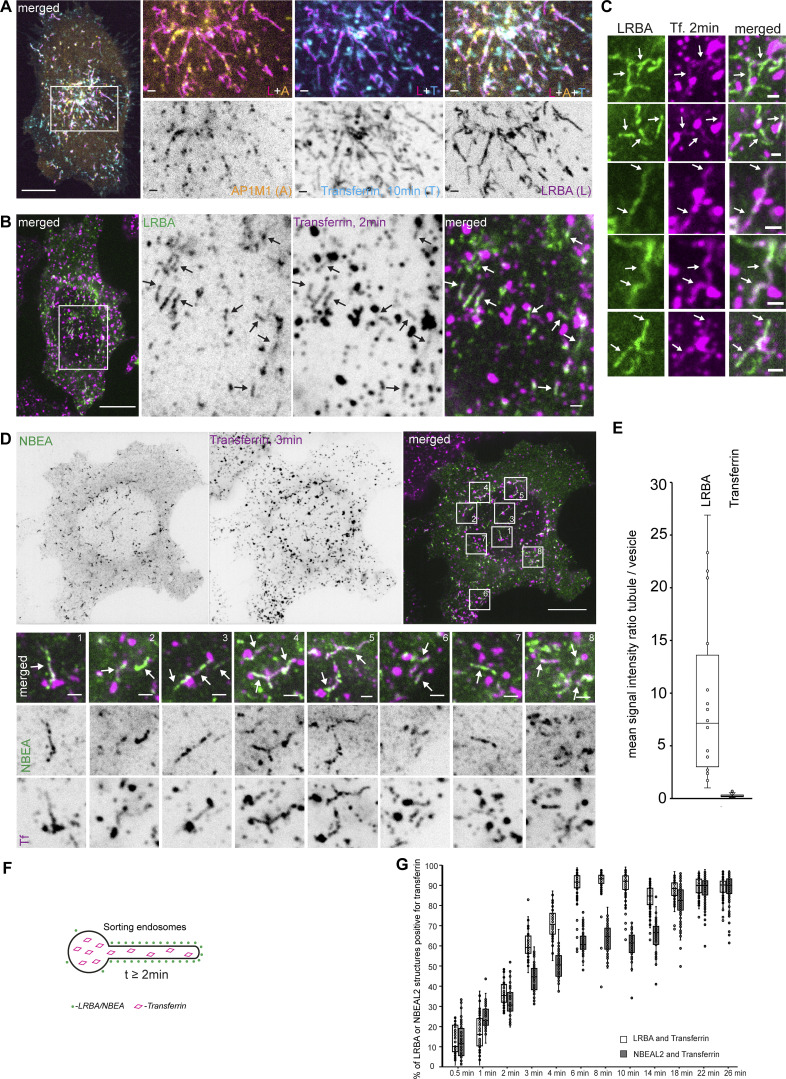
**LRBA- and NBEA- positive tubules accumulate transferrin starting from 2 min after its addition to the culture media. (A)** HeLa cells stably transfected with 3xFlag-EGFP-LRBA and AP1M1-mScarlet were imaged live 10 min after supplementation of growth media with 10 µg/ml of AlexaFluor647-Transferrin. Scale bar 10 µm (main figure) or 1 µm (insert magnifications). **(B)** HeLa cells stably transfected with 3xFlag-EGFP-LRBA were fixed 2 min after supplementation of growth media with 10 µg/ml of AlexaFluor647-Transferrin and imaged by confocal microscopy. Scale bars 10 µm (left merged) or 1 µm (right merged). **(C)** Examples of colocalization of stably transfected 3xFlag-EGFP-LRBA and AlexaFluor647-Transferrin, 2 min after addition of transferrin to the culture media. Scale bars 1 µm. **(D)** HeLa cells stably transfected with 3xFlag-EGFP-NBEA were fixed 3 min after supplementation of growth media with 10 µg/ml of AlexaFluor647-Transferrin and imaged by confocal microscopy. Scale bars 10 µm (left panel) or 1 µm (zoomed-in panels). **(E)** Quantification of the mean signal intensity ratio of LRBA or transferrin on LRBA/transferrin positive tubule vs. nearby LRBA/transferrin positive vesicle. The mean signal intensity of green (endogenous LRBA) or magenta (endocytosed transferrin, 3 min) channels within a 4 × 4 pixels rectangular box over the tubular or vesicular part of the transferrin-positive compartment was used for calculations. *n* = 20. **(F)** Schematic summary of data from Fig. 5, A–E. **(G)** Quantification of the dynamics of colocalization of LRBA or NBEAL2 positive structures and transferrin. HeLa cells stably transfected with 3xFlag-EGFP-LRBA or -NBEAL2 were fixed and imaged at the indicated time points after the addition of 10 µg/ml of AlexaFluor647-Transferrin to the culture media. EGFP or AlexaFluor647 positive structures were segmented and each dot represent the percentage of EGFP-positive objects partially or completely overlapping with AlexaFluor647 positive objects per cell. *n* ≥ 50 per condition.

The observed enrichment of LRBA and NBEA in perinuclear Golgi adjacent tubules containing ARF1 and clathrin coat adaptors indicate that these structures represent the TGN. However, we were surprised to see that the majority of both perinuclear and peripheral LRBA/NBEA and AP1M1 double-positive structures become positive for endocytosed transferrin (Fig. 5 A and Video 4). Transferrin accumulated in LRBA or NBEA-positive tubules starting from 2 min after its addition to the media (Fig. 5, B–D and Fig. S1 G), and by 6 min, 90% of LRBA-positive tubules contained transferrin (Fig. 5 G). Intriguingly, LRBA and NBEA colocalized preferentially with the tubular component of transferrin-positive compartments with little to no LRBA/NBEA staining of transferrin-positive vesicles (Fig. 5, A–F). Thus, at least some LRBA/NBEA and AP1M1 positive tubules are part of the early endocytic/recycling pathway, resembling structures previously described as TSE (Peden et al., 2004) or TEN (Stoorvogel et al., 1996).

**Figure 6.**
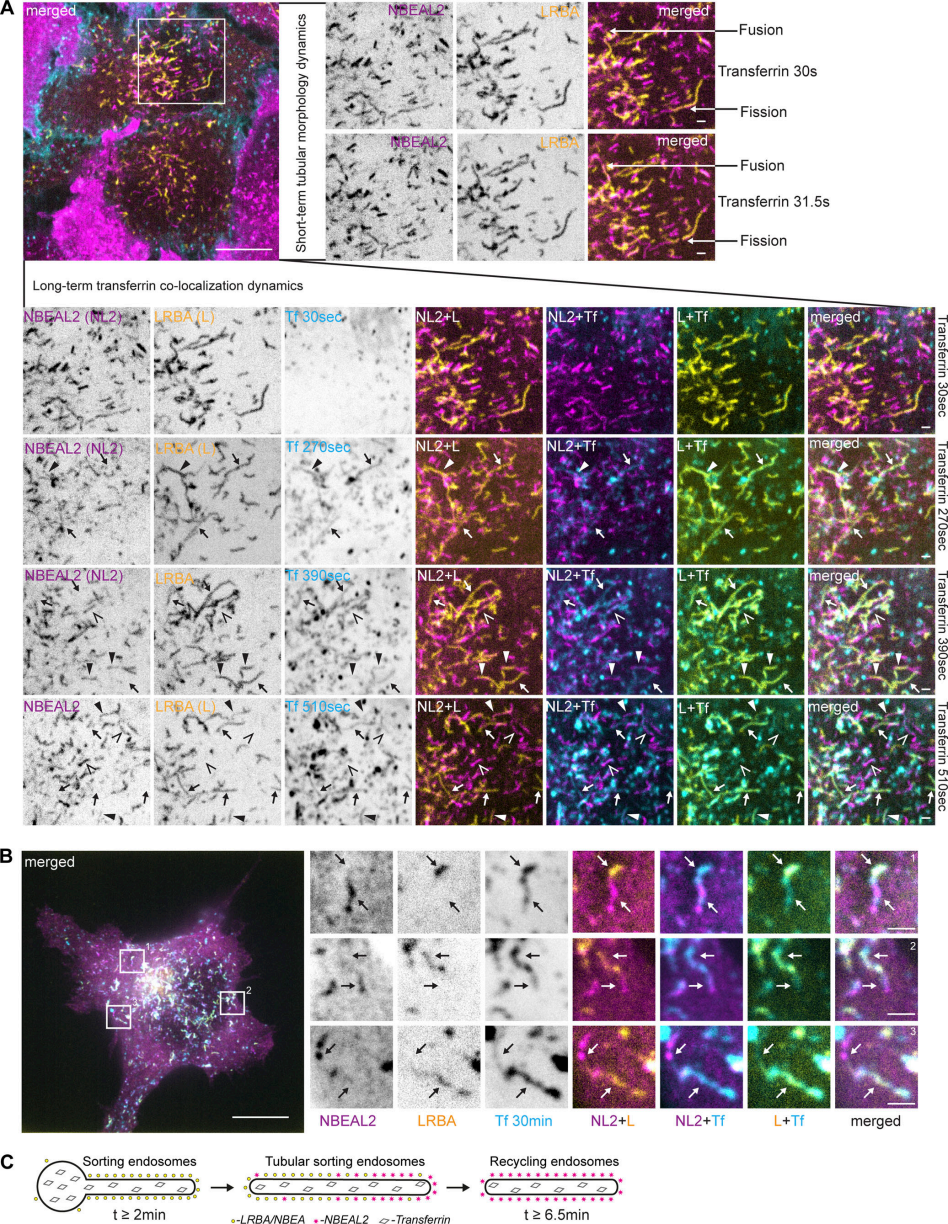
**NBEAL2 shows slower dynamics of colocalization with endocytosed transferrin. (A)** HeLa cells stably transfected with 3xFlag-EGFP-LRBA and mScarlet-NBEAL2 were imaged live with 1-min intervals starting 30 s after supplementation of growth media with 10 µg/ml of AlexaFluor647-Transferrin. Tubules positive for LRBA and NBEAL2 undergo fast fusion or fission events (right zoomed-in inserts). LRBA-positive tubules that accumulate transferrin between 2.5 and 6 min after its addition to the media are either negative (arrow) or only weakly positive for NBEAL2 (solid arrowhead). Tubular structures positive for NBEAL2 and transferrin, but negative for LRBA (open arrowheads), could be observed starting from 6.5 min after the addition of transferrin to the culture media (lower zoomed-in inserts) Scale bars 10 µm (main figure) or 1 µm (inset magnifications). **(B)** HeLa cells stably transfected with 3xFlag-EGFP-NBEAL2 and mScarlet-LRBA were treated for 30 min with 10 µg/ml of AlexaFluor647-transferrin in complete media, fixed, and imaged by confocal microscopy. LRBA and NBEAL2 localize to opposite ends of the same transferrin-positive tubules. Scale bars 10 µm (main figure) or 1 µm (inset magnifications). **(C)** Schematic summary of data from Fig. 6, A and B.

NBEAL2-decorated compartments acquired transferrin with slower dynamics (Fig. 5 G), with transferrin being initially detected in tubules positive only for LRBA and in segments of the tubules positive for both LRBA and NBEAL2 (Fig. 6 A). Localization of transferrin to NBEAL2 positive LRBA negative tubules was only observed from 6 min and 30 s, after the addition of transferrin to the media (Fig. 6 A). We also noticed multiple fission and fusion-like events between LRBA- and NBEAL2-positive compartments (Fig. 6 A, upper panels), with some of the transferrin-positive tubules decorated with LRBA and NBEAL2 on opposite ends (Fig. 6 B). This indicates that transferrin initially passes through the tubules positive for LRBA, followed by its transition through the NBEAL2-decorated compartment (Fig. 6 C).

**Figure 7.**
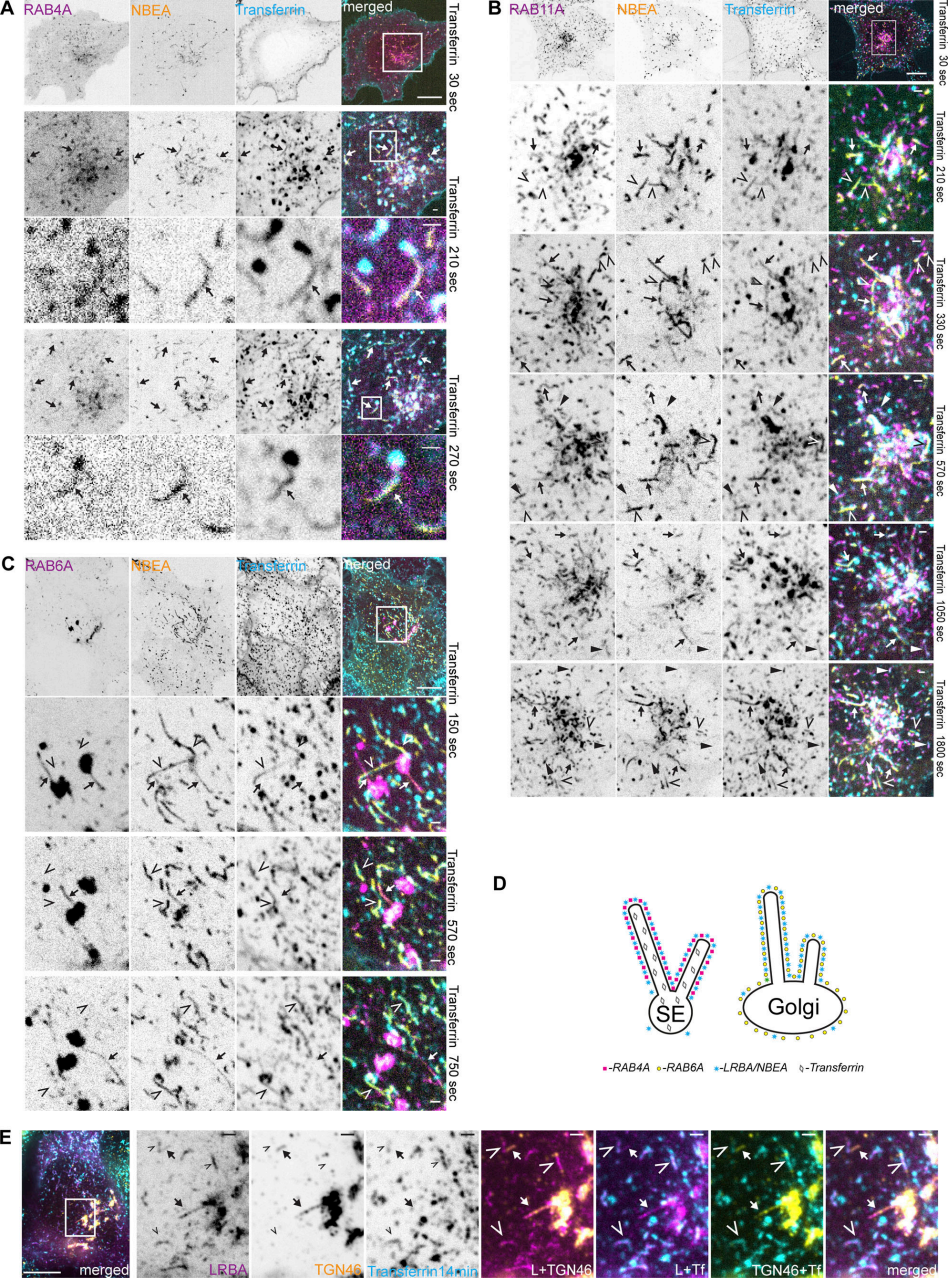
**Transferrin does not accumulate in NBEA-positive tubules decorated with TGN markers RAB6A or TGN46. (A)** HeLa cells stably transfected with 3xFlag-EGFP-NBEA and mScarlet-RAB4A were imaged live with 1 min intervals starting 30 s after supplementation of growth media with 10 µg/ml of AlexaFluor647-Transferrin. Transferrin accumulates in NBEA-positive tubules representing the tubular segment of RAB4A-positive sorting endosomes (arrows). Scale bar 10 µm in the upper row, 1 µm in other panels. **(B)** HeLa cells stably transfected with 3xFlag-EGFP-NBEA and mScarlet-RAB11A imaged live with 1 min intervals starting 30 s after supplementation of growth media with 10 µg/ml of AlexaFluor647-Transferrin. NBEA-positive tubules that accumulate transferrin between 2.5 and 6 min after its addition to the media are either negative (open arrowhead) or only weakly positive for RAB11A (arrow). Starting from 9 min 30 s after addition of transferrin, tubules that are positive for RAB11A and transferrin, but negative for NBEA could be detected (solid arrowhead). Scale bar 10 µm in the upper row, 1 µm in other panels. **(C)** HeLa cells stably transfected with 3xFlag-EGFP-NBEA and mScarlet-RAB6A were imaged live with 1-min intervals starting 30 s after supplementation of growth media with 10 µg/ml of AlexaFluor647-Transferrin. RAB6A-positive NBEA-decorated tubules (arrow) do not accumulate transferrin (arrowhead). Scale bar: 10 µm in the upper row, 1 µm in other panels. **(D)** Schematic summary of data from Fig. 7, A–C. **(E)** HeLa cells stably transfected with 3xFlag-EGFP-LRBA were treated for 14 min with 10 µg/ml of AlexaFluor647-transferrin in complete media, fixed, stained with an anti-TGN46 antibody, and imaged by confocal microscopy. LRBA-decorated tubules, positive for the TGN marker TGN46 (arrow) do not accumulate the endocytic recycling cargo transferrin (arrowhead). Scale bars 10 µm (left merged) or 1 µm (right merged).

To further clarify the nature of the LRBA/NBEA positive compartments, transferrin was internalized in HeLa cells stably expressing NBEA and either RAB4A, RAB6A, or RAB11A, which all partially colocalize with LRBA and NBEA (Table 1; and Fig. 2 D and Fig. S1 E). Live cell imaging demonstrated that NBEA-positive tubules that acquired transferrin early (3 min and 30 s) were positive for RAB4 (Fig. 7 A) and a few were positive for RAB11 (Fig. 7 B), indicating that they are sorting tubules of early/sorting endosomes. Some RAB11A positive and NBEA negative structures acquired transferrin from 9 min 30 s (Fig. 7 B), in line with previous reports of RAB11 being a marker of the slow recycling pathway and delayed dynamics of colocalization between transferrin and RAB11 (Sheff et al., 2002; Ullrich et al., 1996). Upon internalization of transferrin in cells expressing NBEA and RAB6A, we could detect perinuclear tubules that were either positive for RAB6A and NBEA or transferrin and NBEA (Fig. 7, C and D; and Fig. S2 D), and never positive for all three proteins simultaneously. A similar pattern was observed for LRBA-positive tubules, colocalizing with either endogenous TGN46 or transferrin, but not with transferrin and TGN46 simultaneously (Fig. 7 E). Markers of the media- (giantin) and cis-Golgi (GM130) partially overlapped with the perinuclear LRBA signal but showed no colocalization with Golgi-connected LRBA-positive tubules (Fig. S2 E). The lack of endocytosed transferrin in NBEA/RAB6A and LRBA/TGN46 compartments, together with our observation that NBEA/RAB6A and LRBA/TGN46 positive tubules are connected to the Golgi (Figs. 2 D, 3 B, 7 C, S1, E, F, and H; and Fig. S2 D), suggest a secretory nature of these structures and indicate that LRBA/NBEA positive tubules can receive input from both endocytic and secretory pathways.

**Figure S3.**
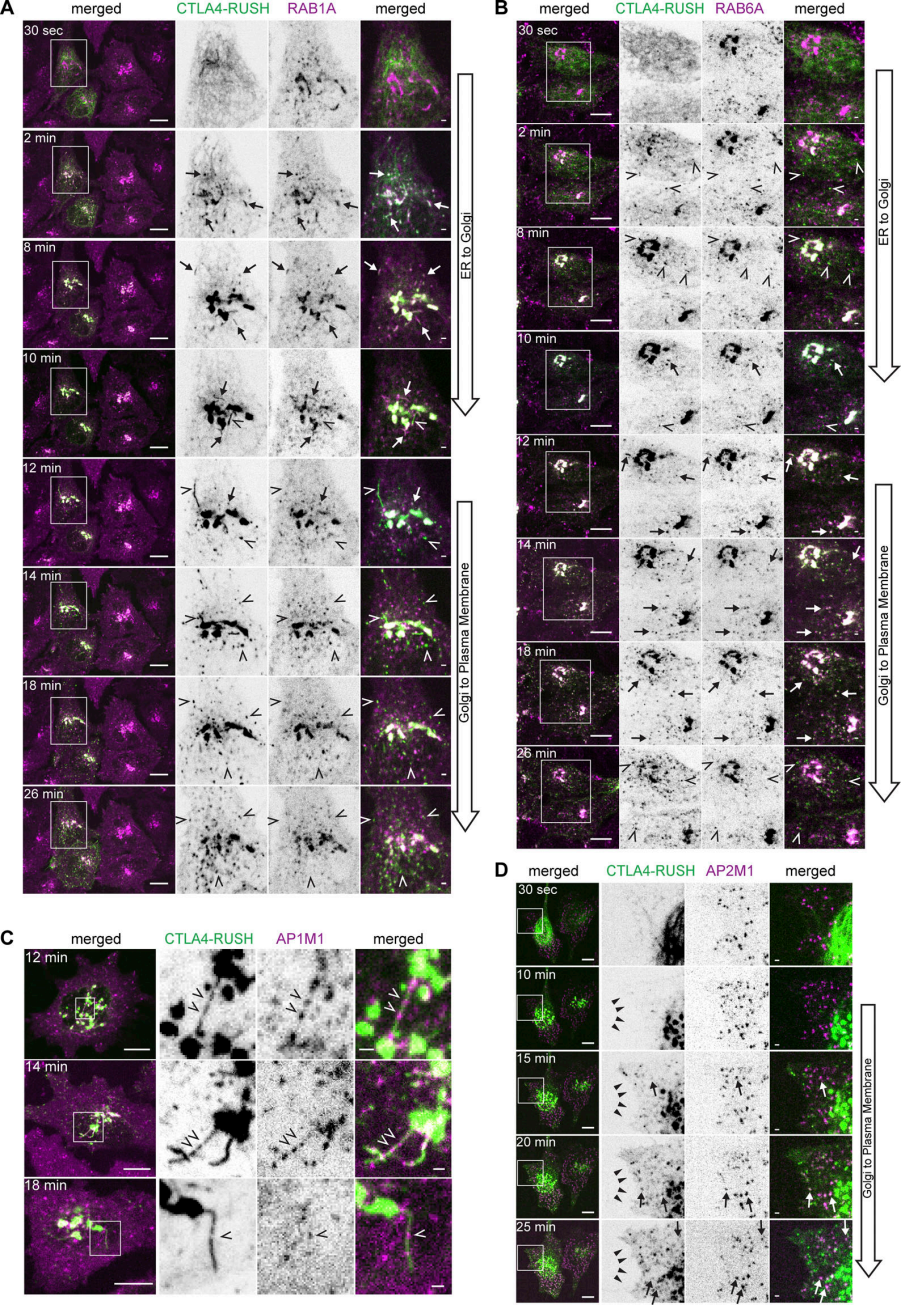
**Characterization of intracellular trafficking of CTLA4-EGFP-RUSH protein. (A)** Live cell imaging of HeLa cells stably transfected with mScarlet-RAB1A and transiently transfected with CTLA4-EGFP-RUSH at the indicated time points after the addition of 50 µM biotin. Arrows and open arrowheads point to colocalization or lack of colocalization of CTLA4 with RAB1A, respectively. Scale bars 10 µm (left merged) or 1 µm (right merged). **(B)** Live cell imaging of HeLa cells stably transfected with mScarlet-RAB6A and transiently transfected with CTLA4-EGFP-RUSH at the indicated time points after the addition of 50 µM of biotin. Arrows and open arrowheads point to colocalization or lack of colocalization of CTLA4 with RAB6A, respectively. Scale bars 10 µm (left merged) or 1 µm (right merged). **(C)** Live cell imaging of HeLa cells stably transfected with AP1M1-mScarlet and transiently transfected with CTLA4-EGFP-RUSH at the indicated time points after the addition of 50 µM of biotin. CTLA4-RUSH is excluded from AP1M1-positive transport carriers forming along the secretory tubule (open arrowhead). Scale bars 10 µm (left merged) or 1 µm (right merged). **(D)** Live cell imaging of HeLa cells stably transfected with AP1M1-mScarlet and transiently transfected with CTLA4-EGFP-RUSH at the indicated time points after the addition of 50 µM of biotin. Arrows point to colocalization of CTLA4 and AP2M1. The cell edge (solid arrowhead) is labeled with CTLA4-RUSH starting from 15 min after initiation of CTLA4 trafficking. Scale bars 10 µm (left merged) or 1 µm (right merged).

**Figure S4.**
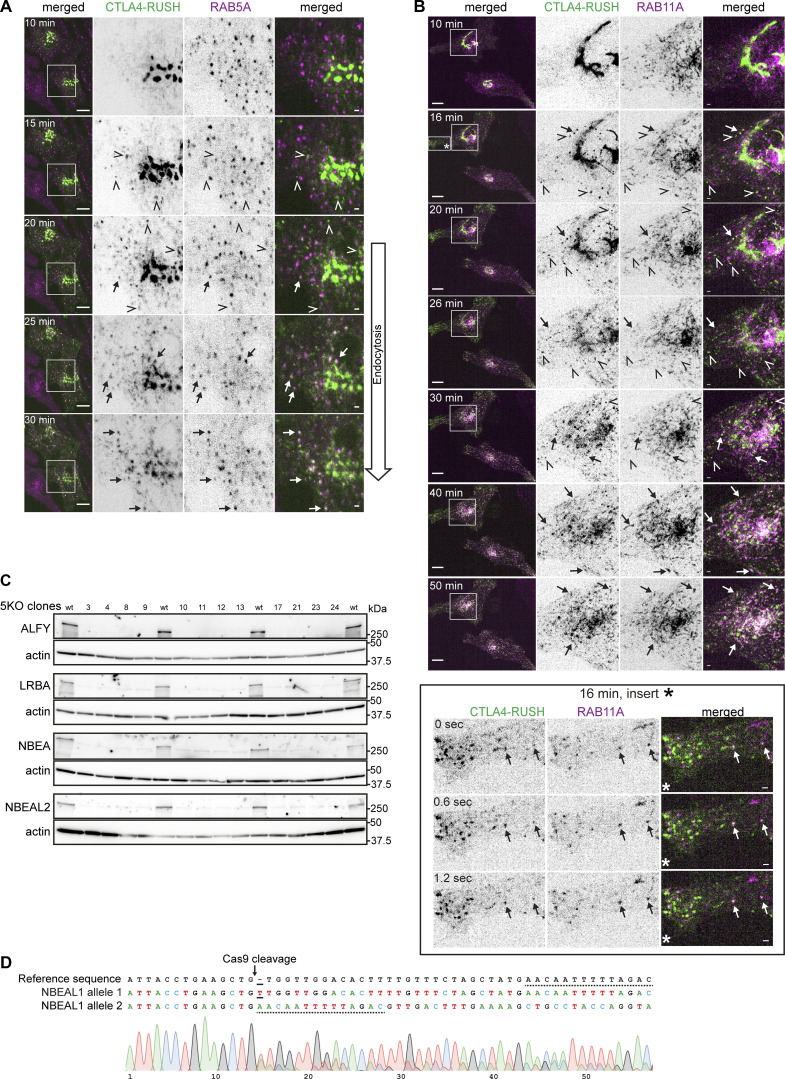
**Characterization of intracellular trafficking of CTLA4-EGFP-RUSH protein and confirmation of BDCPs knockouts. (A)** Live cell imaging of HeLa cells stably transfected with mScarlet-RAB5A and transiently transfected with CTLA4-EGFP-RUSH at the indicated time points after the addition of 50 µM biotin. Arrows and open arrowheads point to colocalization or lack of colocalization of CTLA4 with RAB5A, respectively. Scale bars 10 µm (left merged) or 1 µm (right merged). **(B)** Live cell imaging of HeLa cells stably transfected with mScarlet-RAB11A and transiently transfected with CTLA4-EGFP-RUSH at the indicated time points after the addition of 50 µM biotin. Arrows and open arrowheads point to colocalization or lack of colocalization of CTLA4 with RAB11A, respectively. The box labeled with an asterisk (16 min, insert*) is zoomed in in the lower subpanel to demonstrate colocalization of a fraction of CTLA4 with RAB11A at the 16 min time point. Scale bars 10 µm (left merged) or 1 µm (right merged). **(C)** Western blot of clones of HeLa cells with knockout (5KO) of the reference isoforms of ALFY, LRBA, NBEA, NBEAL1, and NBEAL2. Clones 8 and 12 were renamed as 5KO1 and 5KO2 and used for surface biotinylation experiments. **(D)** Genotyping of HeLa cells with knockout of the reference isoform of NBEAL1. Solid line marks the insertion of a single nucleotide in allele 1, while the dotted line highlights remaining homologous sequence after the deletion of 28 nucleotides in NBEAL1 allele 2. Source data are available for this figure: SourceData FS4.

To further investigate this hypothesis, we performed a Retention Using Selective Hook (RUSH) (Boncompain et al., 2012) assay with CTLA4 as a secretory cargo simultaneously with transferrin internalization in HeLa cells stably expressing LRBA or NBEA. We first assessed the temporary localization of CTLA4-RUSH-EGFP in cells expressing mScarlet-I-RAB1A, -RAB6A, -RAB5A, -RAB11A, -AP1M1 or -AP2M1, demonstrating that CTLA4-RUSH follows conventional secretory and endocytic/recycling pathways that can be subdivided into an early (ER-to-Golgi) and a late (Golgi-to-PM) secretory phase, followed by its endocytosis and recycling (Fig. S3, A–D; and Fig. S4, A and B). Upon addition of biotin to CTLA4-RUSH-EGFP cells, we observed the formation of CTLA4 positive vesicles and tubules that first exclusively colocalized with RAB1A with an increased CTLA4 positive Golgi signal and a decreased ER signal (Fig. S3 A). After 10 min, CTLA4-positive vesicles and Golgi-connected/derived tubules that were negative for RAB1A, but positive for RAB6A were observed (Fig. S3, A and B), suggesting the transport of CTLA4-RUSH-EGFP to late secretory compartments. Interestingly, CTLA4 was excluded from AP1M1 positive puncta that localized along otherwise CTLA4 positive tubules formed during the late secretory phase (Fig. S3 C), suggesting that CTLA4 is secreted from the Golgi in pleomorphic RAB6A-positive carriers and not in CCVs. After 15 min, CTLA4-RUSH-EGFP started to colocalize with AP2M1 positive clathrin-coated puncta and was detected along the cell edge (Fig. S3 D), indicating its arrival at the plasma membrane. After 20 min, we observed colocalization of CTLA4-RUSH-EGFP with RAB5A (Fig. S4 A) in early endosomes. Intriguingly, we observed a partial colocalization of some CTLA4-RUSH positive vesicles with RAB11A already during the secretory phase, suggesting that a fraction of newly synthesized CTLA4 may reach RAB11A positive compartments directly from the Golgi (Fig. S4 B).

**Figure 8.**
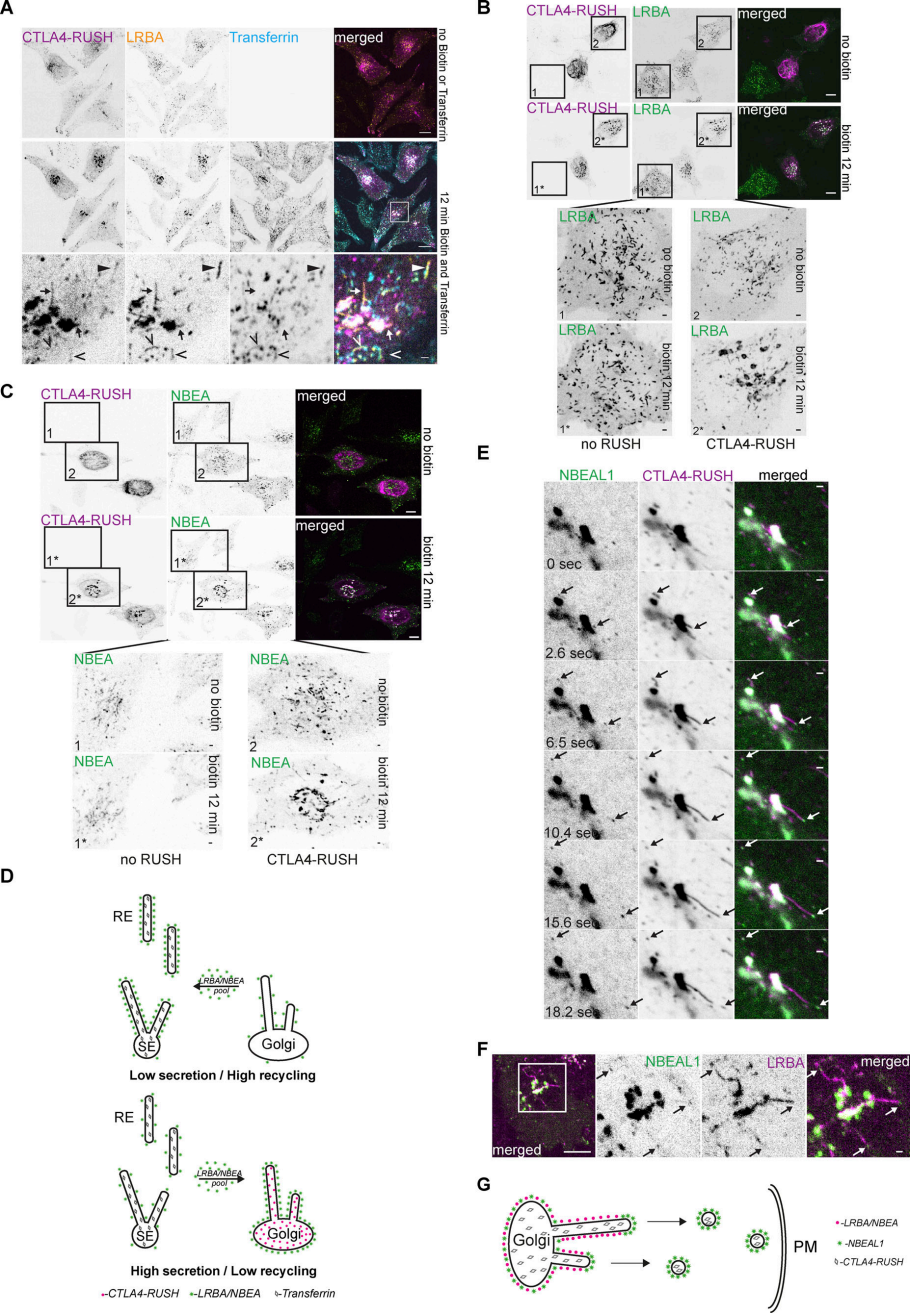
**The influx of secretory cargo redistributes LRBA and NBEA from recycling to secretory compartments and sequester NBEAL1 and LRBA to different subdomains of secretory tubules. (A)** HeLa cells stably transfected with 3xFlag-EGFP-LRBA and transiently transfected with CTLA4-mCherry-RUSH were treated for 12 min with 10 µg/ml of AlexaFluor647-Transferrin and 50 µM of biotin to release transport of CTLA4-mCherry-RUSH from the ER, followed by live imaging before and after the treatment. Golgi-attached LRBA- and CTLA4-RUSH positive secretory tubules lack endocytosed transferrin (arrow) but can acquire transferrin after Golgi detachment (solid arrowhead). LRBA- and transferrin-positive tubules, negative for CTLA4-RUSH are labeled with open arrowheads. Golgi-attached LRBA- and CTLA4-RUSH positive secretory tubules lack endocytosed transferrin (arrow) but can acquire transferrin after Golgi detachment (solid arrowhead). LRBA- and transferrin-positive tubules, negative for CTLA4-RUSH are labeled with open arrowheads. Scale bar 10 µm in the upper and middle panel and 1 µm in the lower panel. **(B)** HeLa cells stably transfected with 3xFlag-EGFP-LRBA and transiently transfected with CTLA4-RUSH-mCherry were imaged before and 12 min after the addition of 50 µM of biotin to the culture media. Numbers represent cells not transfected (1) or transfected (2) with CTLA4-RUSH construct before (1 and 2) or 12 min after (1* and 2*) addition of biotin to the media. Scale bars 10 µm (upper two panels) or 1 µm (lower inserts). **(C)** HeLa cells stably transfected with 3xFlag-EGFP-NBEA and transiently transfected with CTLA4-RUSH construct imaged before and 12 min after the addition of 50 µM of biotin to culture media. Numbers represent cells not transfected (1) or transfected (2) with CTLA4-RUSH construct before (1 and 2) or 12 min after (1* and 2*) addition of biotin to the media. Scale bars 10 µm (upper two panels) or 1 µm (lower inserts). **(D)** Schematic summary of data from Fig. 8, A–C. **(E)** HeLa cells stably transfected with 3xFlag-EGFP-NBEAL1 and transiently transfected with CTLA4-RUSH-mCherry were imaged 12 min after the addition of 50 µM of biotin. NBEAL1 decorates the tip of growing Golgi-connected secretory tubules positive for CTLA4-RUSH-mCherry (arrows) that form transport carriers upon tubular fission. Scale bars 1 µm. **(F)** HeLa cells stably transfected with 3xFlag-EGFP-NBEAL1 and mScarlet-LRBA and transiently transfected with CTLA4-RUSH-mTagBFP2 were imaged live 12 min after the addition of 50 µM of biotin. NBEAL1 and LRBA segregate into different subdomains of Golgi-connected secretory tubules (arrows). mTagBFP2 signal was verified by visual observation, but not imaged, due to the toxicity of the blue-channel imaging setup. Scale bar 10 µm (left merged) or 1 µm (right merged). **(G)** Schematic summary of data from Fig. 8, E and F.

Consistent with our RAB6A/TGN46 colocalization data, we observed that Golgi-connected LRBA-positive tubules could acquire CTLA4-RUSH during the late secretory phase (Fig. 8 A), indicating their secretory nature. These compartments co-existed with LRBA and transferrin-positive and CTLA4-negative tubules, but also with tubules positive for all three proteins, suggesting the mixing of endocytic and secretory cargo in LRBA-positive compartments (Fig. 8 A).

Surprisingly, the addition of biotin to cells co-expressing CTLA4-RUSH-EGFP and LRBA or NBEA caused a redistribution of not only CTLA4 to the Golgi but of LRBA and NBEA from cytosolic tubular structures to Golgi stacks (Fig. 8, A–C). This was not observed in cells expressing only LRBA or NBEA (Fig. 8, B and C), suggesting that their redistribution is caused by the excess influx of CTLA4-RUSH as a secretory cargo into the Golgi (Fig. 8 D).

When carrying out the CTLA4-RUSH assay in HeLa cells stably expressing NBEAL1, we observed an enrichment of NBEAL1 signal on the tip of growing secretory tubules containing CTLA4-RUSH 12 min after the addition of biotin. Detachment of NBEAL1-positive tubular tips generated CTLA4- and NBEAL1-positive vesicles that moved toward the plasma membrane (Fig. 8 E and Video 5). Intriguingly, imaging of HeLa cells co-expressing 3xFlag-EGFP-NBEAL1, mScarlet-LRBA, and CTLA4-RUSH-mTagBFP2 revealed that NBEAL1 were enriched on the tip, while LRBA distributed along the body of the same tubule (Fig. 8, F and G), and that transport carriers formed upon tubular fission preferentially contained either LRBA or NBEAL1 (Video 6).

**Video 5.** **NBEAL1 decorates the tip of Golgi-connected secretory tubules positive for CTLA4-RUSH-mCherry (arrows).** HeLa cells stably transfected with 3xFlag-EGFP-NBEAL1 and transiently transfected with CTLA4-RUSH-mCherry were imaged 12 min after the addition of 50 µM of biotin. Scale bars 10 µm. Playback speed 18x.

**Video 6.** **NBEAL1 and LRBA segregate into different subdomains of Golgi-connected secretory tubules (arrows).** HeLa cells stably transfected with 3xFlag-EGFP-NBEAL1 and mScarlet-LRBA and transiently transfected with CTLA4-RUSH-mTagBFP2 were imaged live 12 min after the addition of 50 µM of biotin. mTagBFP2 signal was verified by visual observation, but not imaged, due to the toxicity of the blue-channel imaging setup. Scale bar 10 µm. Playback speed 25x.

In summary, our data show that LRBA and NBEA localize both to secretory tubules of the TGN and to sorting tubules of early/sorting endosomes and that they can redistribute between these compartments upon the influx of secretory cargo. Moreover, we showed that LRBA and NBEAL1 can segregate either to the tip (NBEAL1) or the body (LRBA) of the same secretory tubule and generate two distinct transport carriers upon fission of the tubule. Similarly, we demonstrated that LRBA and NBEAL2 can segregate to different ends of transferrin-positive recycling tubules, suggesting an analogous mechanism for the generation of endocytic recycling carriers.

### BDCPs function as protocoatomer proteins in the sorting of transmembrane secretory and endocytic cargo

The data presented above, combined with the presence of a large extended alpha solenoid and a beta-propeller domain in all typical BDCPs, suggests that these proteins may have a protocoatomer origin and function as membrane coat proteins (Rout and Field, 2017). This hypothesis is further supported by the reported mis-sorting of several TMPs upon the loss of function of LRBA or NBEA (Gromova et al., 2018; Lo et al., 2015; Nair et al., 2013). Moreover, LRBA was previously suggested to bind to and compete with AP1 for binding to a YVKM motif in the cytosolic tail of CTLA4 (Lo et al., 2015).

**Figure 9.**
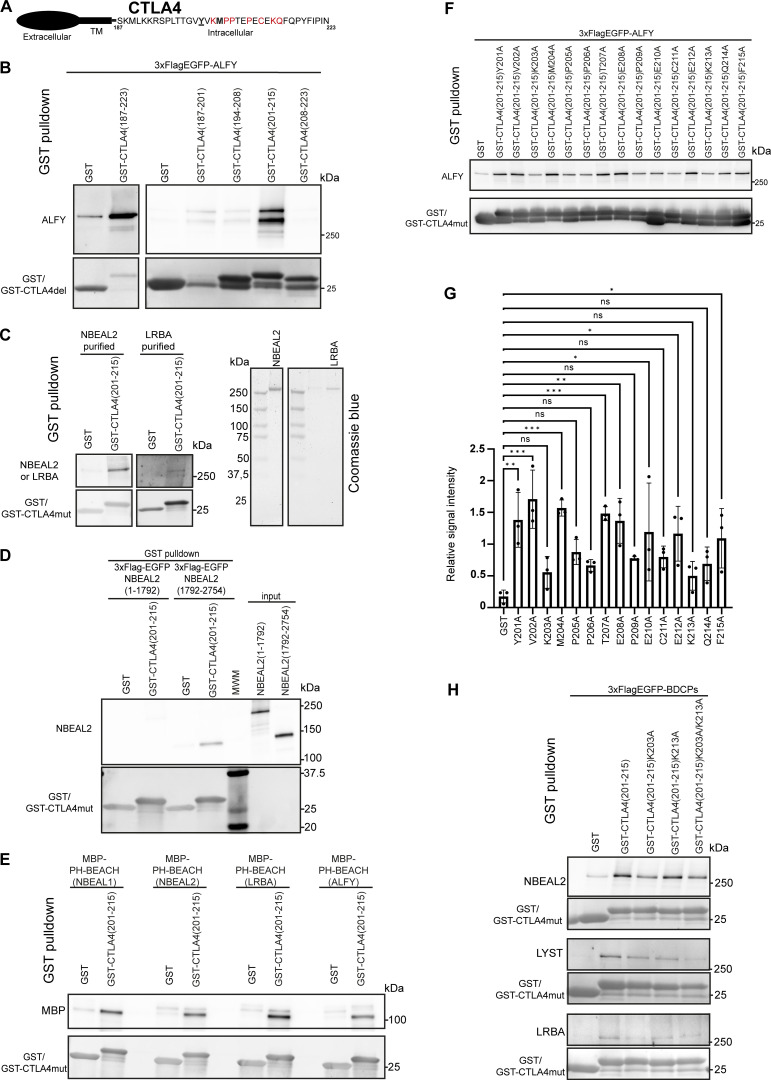
**BDCPs bind to the cytosolic tail of CTLA4. (A)** Schematic structure of CTLA4 with the amino acid sequence of the cytosolic tail indicated. Bold residues indicate the YxxM AP1-binding motif with underlined Y201 previously reported as being essential for binding to LRBA. Amino acids shown in red were identified as important for binding to ALFY in the current study. **(B)** ALFY interacts with aa 201–215 in the cytosolic tail of CTLA4. GST-tagged fragments of CTLA4 immobilized on glutathione-sepharose beads were incubated with lysate of HeLa cells stably transfected with 3xFlag-EGFP-ALFY. Bound 3xFlag-EGFP-ALFY were detected by staining with an anti-flag antibody, while GST-tagged proteins were detected by staining with ponceau S. **(C)** Purified NBEAL2 and LRBA (right panels) can bind to the cytosolic tail of CTLA4. Purified full-length untagged NBEAL2 or LRBA were incubated with GST-tagged CTLA4 (201–215) immobilized on glutathione-sepharose beads. Bound NBEAL2 or LRBA were detected by staining with anti-NBEAL2 or anti-LRBA antibodies, while GST-tagged proteins were detected by staining with an anti-GST antibody. **(D)** The C-terminal fragment of NBEAL2 interacts with the cytosolic tail of CTLA4. Purified N- (aa 1–1792) or C- (aa 1792–2794) terminal fragments of NBEAL2 fused to 3xFlagEGFP were incubated with GST-tagged CTLA4 (201–215) immobilized on glutathione-sepharose beads. Bound 3xFlag-EGFP-NBEAL2 was detected with an anti-Flag antibody, while GST-tagged proteins were detected by staining with an anti-GST antibody. **(E)** The PH-BEACH domains of NBEAL1, NBEAL2, LRBA, and ALFY bind to the cytosolic tail of CTLA4. Purified PH-BEACH domains of NBEAL1, NBEAL2, LRBA, and ALFY fused to MBP were incubated with GST-tagged CTLA4 (201–215) immobilized on glutathione-sepharose beads. Bound MBP-fusion proteins were detected with an anti-MBP antibody, while GST-tagged proteins were detected by staining with an anti-GST antibody. **(F)** Alanine scan mutagenesis of the ALFY-binding region of CTLA4 (aa 201–215). GST-CTLA4 (201–215) point mutants were immobilized on glutathione-sepharose beads and incubated with HeLa 3xFlag-EGFP-ALFY cell lysate. Bound 3xFlag-EGFP-ALFY were detected by staining with an anti-flag antibody, while GST-tagged proteins were detected by staining with an anti-GST antibody. **(G)** Quantification of data in F from three independent experiments. Values shown are mean ± SD ***P < 0.001, **P < 0.01, *P < 0.05, one-way ANOVA. **(H)** Binding of several BDCPs to the cytosolic tail of CTLA4. Wild type or mutant GST-CTLA4(201–215) were immobilized on glutathione-sepharose beads and incubated with lysate from HeLa cells overexpressing 3xFlag-EGFP-NBEAL2, - LYST or -LRBA. Bound proteins were detected by staining with an anti-flag antibody, while GST-tagged proteins were detected by staining with an anti-GST antibody. Source data are available for this figure: SourceData F9.

To address whether BDCPs may bind to the cytosolic tails of TMPs and function as TMPs sorting adaptors, we performed pulldown assays with the cytosolic tail of CTLA4 fused to GST and cell lysates from Hela cells expressing different BDCPs as 3xFlag-EGFP fusion proteins (Fig. 9, A, B, and H) or purified full-length NBEAL2 and LRBA (Fig. 9 C). This revealed that several BDCPs (LRBA, LYST, NBEAL2, and ALFY) bind to the cytosolic tail of CTLA4 (Fig. 9, B, C, and H). We further mapped the binding specificity of 3xFlag-EGFP-ALFY to the cytosolic tail of CTLA4 (aa 187–223), showing that CTLA4 aa 201–215 was sufficient for binding (Fig. 9, B and H). To map the region of BDCPs important for binding, GST-CTLA4 (201–215) was incubated with purified N-terminal (α-solenoid and ConA-like domains) or C-terminal (PH-BEACH-WD40 domains) fragments of NBEAL2, demonstrating binding of the C-terminal region to the cytosolic tail of CTLA4 (Fig. 9 D). Intriguingly, the purified PH-BEACH domain assembly of NBEAL1, NBEAL2, LRBA, and ALFY all bound to GST-CTLA4 (201–215), indicating that the PH-BEACH domain of BDCPs interacted with cytosolic tails (Fig. 9 E). An alanine scan of CTLA4 (201–215) identified lysine-203 and -213 and proline-205, -206 and -209, to be essential for the interaction with ALFY, while tyrosine-201 and methionine-204 in the 201-YVKM-204 motif, previously reported to interact with LRBA and the AP1 clathrin coat (Lo et al., 2015; Schneider et al., 1999), were dispensable for binding to ALFY (Fig. 9, F and G). We further confirmed the importance of lysine-203 and -213 in GST pulldown assays with other BDCPs, including LRBA, LYST, and NBEAL2 (Fig. 9 H). The binding site of BDCPs in the cytosolic tail of CTLA4 overlaps with the reported AP1M1 and AP2M1 binding sites (Schneider et al., 1999), indicating a possible competition between the two. This, combined with the observed sequestration of several BDCPs and clathrin coat adaptors to different subdomains of secretory or recycling membrane tubules, followed by formation of transport carriers upon tubular fission indicate that BDCPs function as competitive adaptors for transmembrane cargo sorting.

**Figure 10.**
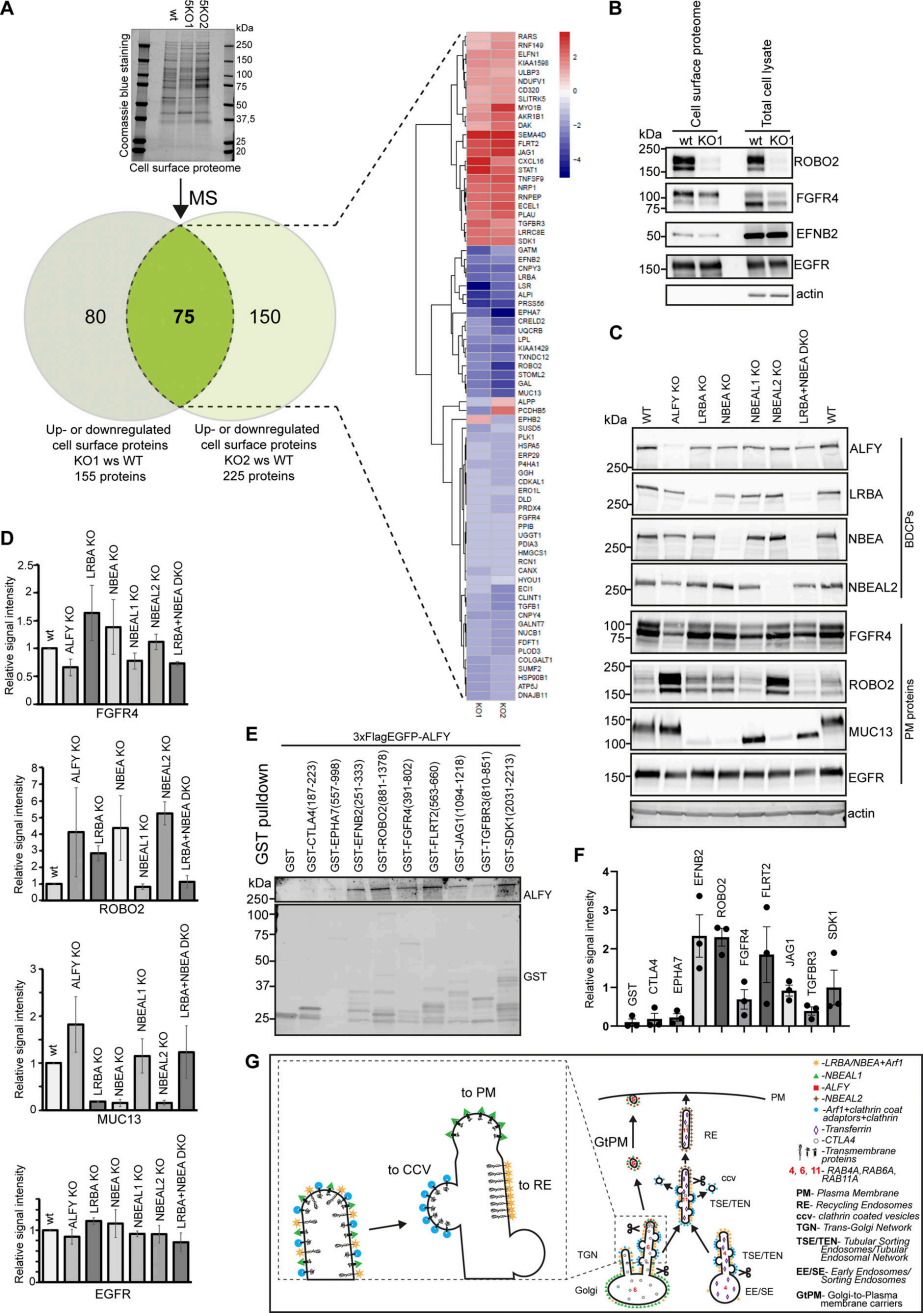
**Knockout of reference isoforms of BDCPs leads to missorting of plasma membrane proteins. (A)** The plasma membrane proteome of wild-type HeLa cells and two clones of HeLa cells lacking the reference isoforms of ALFY, LRBA, NBEA, NBEAL1, and NBEAL2 (KO1 and KO2) were isolated and analyzed by mass spectrometry. The Venn diagram and heatmap depict proteins that were significantly up- or downregulated in knock-out clones compared with wild-type cells. **(B)** Western blot of the indicated proteins from the cell surface proteome and total cell lysates of wild type Hela cells and HeLa cells lacking reference isoforms of ALFY, LRBA, NBEA, NBEAL1, and NBEAL2 (KO1). **(C)** Knockout of single BDCPs differentially affects the level of proteins identified as up or down-regulated on the plasma membrane of 5KO cells. Western blot of the indicated proteins from total cell lysates of wild-type HeLa and HeLa cells lacking reference isoforms of either ALFY, LRBA, NBEA, NBEAL1, or NBEAL2. **(D)** Quantification of data in C from three independent experiments. Values shown are mean ± SD. **(E)** Binding of ALFY to cytosolic tails of selected transmembrane proteins found to be up- or down-regulated in BDCPs 5KO cells. GST-tagged cytosolic tails of CTLA4, EPHA7, EFNB2, ROBO2, FGFR4, FLRT2, JAG1, TGFBR3, or SDK1 were immobilized on glutathione-sepharose beads and incubated with HeLa 3xFlag-EGFP-ALFY cell lysate. Bound proteins were detected by staining with an anti-flag antibody, while GST-tagged proteins were detected by staining with an anti-GST antibody. **(F)** Quantification of data in C from three independent experiments. Values shown are mean ± SD. **(G)** Schematic model of BDCPs function as transmembrane cargo sorting adaptors on trans-Golgi network and tubular sorting endosomes/tubular endosomal network. Source data are available for this figure: SourceData F10.

To identify novel TMPs that rely on BDCPs for their normal cellular trafficking, we used CRISPR/Cas9 to knock out the reference isoforms of five BDCPs (5 KO) localizing in a partial or completely overlapping manner on secretory or recycling compartments (ALFY, LRBA, NBEA, NBEAL1, and NBEAL2) in HeLa cells to minimize potential redundancy effect (Fig. S4, C and D). Wild type (wt) and 5KO cells were subjected to surface biotinylation and quantitative mass spectrometry of the plasma membrane proteome. We identified 48 proteins that were significantly downregulated and 24 proteins that were significantly upregulated on the plasma membrane of two clones of the 5KO cells compared to wt cells (Fig. 10 A). The downregulation of selected proteins (ROBO2, EFNB2, and FGFR4) at the plasma membrane was confirmed by western blot analysis (Fig. 10 B), while the expression of other proteins (as EGFR) was unaffected (Fig. 10, A and B). Importantly, the effect of knocking out single BDCPs on the level of these identified transmembrane hits was not uniform (Fig. 10, C and D), indicating that the different BDCPs regulate trafficking of distinct TMPs. To check whether the identified down or upregulated proteins can indeed bind to BDCPs, we cloned the cytosolic tails of eight selected proteins (EPHA7, EFNB2, ROBO2, FGFR4, FLRT2, JAG1, TGFBR3, and SDK1) as GST fusion proteins and tested their binding to purified 3xFlag-EGFP-ALFY (Fig. 10, E and F). Intriguingly, the cytosolic tails of all tested proteins bind to ALFY, suggesting that their plasma membrane trafficking defect originates from loss of binding to BDCPs.

## Discussion

We here present the first systematic study of the human BDCP family. AlfaFold structure model predictions show that typical BDCPs all share an alpha-solenoid/beta-propeller molecular structure, indicating they may have a protocoatomer origin and function as membrane coat proteins. We have cloned, expressed, and mapped the subcellular localization of seven human BDCPs based on their dynamic colocalization with co-expressed RAB proteins, demonstrating their localization to the Golgi and Golgi-to-PM anterograde and retrograde carriers (NBEAL1), peripheral secretory and early endosomal vesicles (ALFY), early/sorting endosomes (NSMAF), late endosomes/lysosomes (LYST), recycling endosomes (NBEAL2), and the TGN/TSE (LRBA and NBEA). Moreover, we showed that BDCPs and specific clathrin coat adaptors localize to distinct subdomains of post-Golgi secretory and endocytic recycling tubules in a beads-on-a-string-like manner. We demonstrate that BDCPs interact directly with the cytosolic tail of selected TMPs and that depletion of BDCPs results in the mis-sorting of several TMPs to the plasma membrane. Thus, we propose a novel function for BDCPs as membrane coat proteins that bind to the cytosolic tails of TMPs at distinct subdomains of post-Golgi secretory and endocytic recycling tubules to regulate their sorting into Golgi-to-PM carriers, CCVs, and RAB11A-positive secretory/recycling compartments.

Our study also highlights the similarity of protein composition and morphology of the TGN and the TSE as dynamic pools of tubular compartments positive for LRBA, NBEA, ARF1, clathrin coat adaptors, and clathrin heavy chain. Our data demonstrate that after fission from RAB6A-positive secretory or RAB4A-positive endocytic compartments, LRBA/NBEA/ARF1/clathrin coat positive tubules lose the original RAB GTPase and acquire RAB11A. In *S. cerevisiae*, such a transition is mediated by a Rab GAP cascade (Suda et al., 2013) and possibly Arf1 activity (Thomas et al., 2021). These compartments undergo luminal and membranous content mixing due to tubular fusion, followed by content partitioning into separate carriers due to the budding of CCVs and fission of tubules themselves. The crosstalk between these compartments of secretory or endocytic origin also manifests in the form of redistribution of LRBA and NBEA from recycling tubules to Golgi and Golgi-derived secretory tubules upon the influx of secretory cargo, which could indicate a regulatory feedback mechanism. Thus LRBA/NBEA-positive tubules resemble the plant TGN/EE structures, functioning as a sorting hub that receives, classifies, and reroutes both secretory and endocytic cargo to their final destination (Nakano, 2022).

We speculate that competitive binding to the cytosolic tails of TMPs of either BDCPs or clathrin coat adaptors, as was suggested for LRBA and AP1M1 binding to the cytosolic tail of CTLA4 (Lo et al., 2015; Schneider et al., 1999), followed by partition of transmembrane cargo into either clathrin-coated buds, interbudding tubular zones positive for LRBA/NBEA, or tubular tips, positive for NBEAL1, precede the generation of transport carriers (Fig. 10 G). Our study suggests that PM-destined secretory or recycling cargo that passes the filter of LRBA/NBEA/ARF1/clathrin coat sorting tubules retains in NBEAL1/RAB6A- or NBEAL2/RAB11-positive tubular segments devoid of ARF1 and clathrin coat adaptors and become transported to the PM after fission of these tubular regions (Fig. 10 G).

The molecular marker composition of LRBA/NBEA positive tubules of secretory origin also resembles those of immature secretory granules, which are also decorated with ARF1 GTPase, AP1, and the GGA family of clathrin coat adaptors (Austin et al., 2000; Bonnemaison et al., 2013; Burgess et al., 2011; Dittié et al., 1996; Lui-Roberts et al., 2005; Tooze and Tooze, 1986). Common for both compartments is the transitioning from RAB6 to RAB11 as a dominant surface RAB GTPase and loss of clathrin coat upon their maturation (Ma and Brill, 2021; Tooze, 1991; Wells et al., 2023, *Preprint*). Clathrin-coated buds on immature secretory granules are believed to sort out unwanted proteins according to a “sorting-by-retention” mechanism (Tooze, 1998), suggesting their similar function in LRBA/NBEA-positive tubules. The presence of luminal cargo of both endocytic and secretory origin is a common feature of lysosome-related organelles (LRO) — the other compartment of the regulated secretory pathway (Delevoye et al., 2019). Loss of function mutations or knockout of several BDCPs manifested with defects in the formation of secretory granules or LRO, including platelets alpha granules for NBEAL2 (Lo et al., 2018), CTLA4-containing compartments for LRBA (Lo et al., 2015), large dense core vesicles and small synaptic vesicles for NBEA (Castermans et al., 2010; Medrihan et al., 2009), and platelet dense granules and melanosomes for LYST (Introne et al., 1999).

Both RAB6 and RAB11 are known molecular markers of transport carriers destined for PM of either secretory or endocytic origin (Chen et al., 1998; Fourriere et al., 2019; Green et al., 1997; Grigoriev et al., 2007; Lock and Stow, 2005). The dynamics of secretory NBEAL1/RAB6A-positive vesicles formation from the pinching of the tip of Golgi-connected secretory tubules, observed in the current study, conceptually resembles the previously described generation of direct Golgi-to-PM carriers (Fourriere et al., 2019; Hirschberg et al., 1998). These carriers preserve RAB6A but loose the ARF1 coat. Perinuclear compartments labeled with mammalian RAB11 are generally described as slow-recycling endosomes (Ullrich et al., 1996). RAB11 homologs in yeast (Ypt31/32), plants (RabA family), and protozoal alveolate *Toxoplasma gondii* however decorate secretory vesicles (Pang et al., 2022; Venugopal et al., 2020) and are essential for the constitutive secretory pathway. In the current study, we demonstrate that a minor fraction of CTLA4-RUSH cargo colocalizes with RAB11A soon after leaving the Golgi. This is in line with previous studies showing that secretory cargo passes through RAB11 compartments on their way from the Golgi to the plasma membrane (Chen et al., 1998; Khvotchev et al., 2003; Lock and Stow, 2005), implying RAB11 as a marker of indirect secretory carriers. RAB11- and NBEAL2-decorated tubules and vesicles are devoid of ARF1 GTPase or AP1M1 clathrin coat adaptor. They acquire endocytosed transferrin later than tubules positive for LRBA/NBEA/ARF1 and clathrin, with NBEAL2 and LRBA occasionally decorating different ends of the same transferrin-positive tubules. We propose that NBEAL2/RAB11-positive compartments are functionally similar to NBEAL1/RAB6 Golgi-to-PM carriers, as transport vesicles that mature from LRBA/NBEA/ARF1/clathrin coat buds compartments and incorporate proteins sorted out from LRBA/NBEA tubules to the plasma membrane (Fig. 10 G).

Our study suggests that the majority of the structures labeled with the AP1M1 clathrin coat adaptor, resolvable by light microscopy, represent clathrin-coated buds and not detached vesicles. Several studies of the TGN and TGN-derived carriers by EM tomography (Ladinsky et al., 1994, 1999; Polishchuk et al., 2006) or light microscopy (Stockhammer et al., 2023, *Preprint*; Bottanelli et al., 2017) support our findings. A recent report describes ARF1-positive tubular compartments containing AP1 and AP3 clathrin coat adaptors and clathrin as beads-on-a-string that function as hubs for short-range cargo transfer to endosomes (Stockhammer et al., 2023, *Preprint*). The authors suggest that such ARF1/clathrin-positive tubules coordinate both Golgi export and endocytic recycling, in line with the LRBA/NBEA/ARF1/clathrin-positive tubules described in our study. Moreover, the reports of a relatively slow assembly speed of AP2 and the clathrin coat on the plasma membrane, followed by fast uncoating dynamics of pinched CCVs (Ehrlich et al., 2004; Lee et al., 2006; Massol et al., 2006) imply the preferential localization of clathrin coat adaptors to donor membrane compartments and not to mature coated vesicles. Interestingly, we often observed that LRBA/NBEA tubular fissions occurred at or close to AP1M1-positive speckles. In line with this, disruption of AP1-positive CCV formation by treatment with Brefeldin A or siRNA-mediated downregulation of clathrin heavy chain results in long tubules, originating from Golgi or endosomes (Hooy et al., 2022; Lippincott-Schwartz et al., 1991; Wood et al., 1991). It is tempting to speculate that pinching of CCV also facilitates the fission of the donor tubular compartment, either by creating a mechanically weak spot along the length of the tubule or by recruiting dynamins not only to the neck of pinching CCV but also to the donor tubule itself. A more careful interpretation of knockdown/knockout phenotypes of CCV machinery components is needed to conclude whether they are also required for the formation of pleomorphic tubulovesicular carriers.

We show that LYST, the largest BDCP, localizes to late endosomes and lysosomes, reflecting its known loss of function phenotype of enlarged lysosomes and LRO (Introne et al., 1999; White, 1966). It was recently found that LYST-deficient human neurons exhibit hyperelongated tubules extruding from enlarged autolysosomes and suggested that defect autophagic lysosome reformation was causing this phenotype (Serra-Vinardell et al., 2023). Given our data on the function of other typical BDCPs, it is tempting to speculate that LYST functions as a cargo adaptor for TMPs together with lysosomal AP2 or AP3 on lysosomal reformation tubules.

In addition to its previously reported localization to RAB5A-positive early endosomes (Søreng et al., 2022), we identified ALFY on peripherally localized highly dynamic RAB6-positive secretory carriers and RAB8-positive tubules, reportedly of either secretory (Dennis et al., 2023, *Preprint*) or recycling (Rangaraj et al., 2023, *Preprint*) origin. ALFY and RAB6-positive vesicles accumulated close to the plasma membrane in filopodia-like protrusions at leading and lagging cellular edges or were observed as a fast circularly moving population of vesicles, migrating along the cellular edge between neighboring cell protrusions. Colocalization of ALFY with peripheral RAB5, RAB6, and RAB8 compartments suggests its possible involvement in cycles of peripheral secretion, endocytosis, and recycling to modulate the local protein landscape of the plasma membrane. Interestingly, ROBO2, EPHA7, FLRT2, and SDK1 identified in this study as plasma membrane proteins that were up- or downregulated in HeLa cells lacking five BDCPs and as direct binders of ALFY are known mediators of neuronal cell migration and axonal guidance, the process reportedly defective in ALFY KO mice and cell culture models (Dragich et al., 2016; Søreng et al., 2022).

Reported loss of function phenotypes of LRBA, NBEA, NBEAL2, ALFY, and WDFY4 are limited to specific cell or tissue types where the mutated proteins are highly expressed, preferentially affecting the CNS (ALFY and NBEA) (Le Duc et al., 2019; Mulhern et al., 2018), immune system (LRBA and WDFY4) (Lopez-Herrera et al., 2012; Theisen et al., 2018), or platelets/megacaryocytes (NBEAL2) (Kahr et al., 2011). We suggest that the relatively high mutational robustness of BDCPs is likely due to several levels of functional redundancy. While typical BDCPs are evolutionarily conserved and can be found in all branches of eukaryotes, the mammalian BDCP family underwent a recent gene duplication event during the evolutionary transition from cephalochordates to vertebrates, generating gene pairs LRBA/NBEA, NBEAL1/NBEAL2, and ALFY/WDFY4. Our study identified LRBA and NBEA in the same compartment, while NBEAL1 and NBEAL2 were found in distinct, but functionally similar compartments, hinting at a possible functional overlap. Around two-third of the length of typical BDCPs constitute a predicted large alpha solenoid domain, consisting of tandem HEAT or Armadillo repeats, encoded by 25–35 exons. The modular nature of tandem repeat alpha-solenoid structures (Reichen et al., 2014; Scholze and Boch, 2011) and the presence of numerous splice isoforms of BDCPs lacking one or several tandem repeats (Bindesbøll et al., 2020) suggest a possible resilience of these proteins to nonsense or missense mutations within the alpha-solenoid due to functional compensation by alternatively spliced isoforms.

In summary, our study demonstrates a common function for typical BDCPS as novel TMP cargo adaptors of protocoatomer origin. We propose a novel mechanism of TMP sorting at the TGN and TSE involving the competitive binding of BDCPs and clathrin coat adaptors to the cytosolic tails of TMPs, followed by their partitioning in distinct subdomains along secretory or recycling tubules and subsequent tubular fission leading to the formation of specific transport vesicles.

## Materials and methods

Antibodies are listed in Table 2. Bacterial and virus strains are listed in Table 3. Chemicals, peptides, and recombinant proteins are listed in Table 4. Experimental models: Cell lines are listed in Table 5. Recombinant DNA is listed in Table 6.

**Table 2.** Antibodies

	Source	Identifier
LAMP1 monoclonal (clone H4A3) antibody	Santa Cruz Biotechnology	Cat# sc-20011
EEA1 monoclonal (clone 14/EEA1) antibody	BD Biosciences	Cat# 610457
ROBO2 monoclona (clone E4M6D) antibody	Cell Signaling Technology	Cat# 45568
FGFR4 monoclonal (clone D3B12) antibody	Cell signaling Technology	Cat# 8562S
EFNB2 monoclonal (clone JM53-21) antibody	Thermo Fisher Scientific	Cat# MA5-32740
EGFR antibody	Fitzgerald Industries	Cat# 20-ES04
MUC13 antibody	Cell Signaling Technology	Cat# 44454
Beta-actin monoclonal (clone 8H10D10) antibody	Cell Signaling Technology	Cat# 3700S
Flag-tag monoclonal (clone M2) antibody	Sigma-Aldrich	Cat# F1804-200UG
GST monoclonal (clone 3G10/1B3) antibody	Abcam	ab92
ALFY monoclonal antibody	Ai Yamamoto Lab, Fox et al. (2020)	NA
LRBA antibody	Atlas Antibodies	Cat# HPA019366
LRBA antibody	Atlas Antibodies	Cat# HPA023597
NBEA antibody	Novus Biologicals	Cat# NBP1-90004
NBEAL2 monoclonal (clone EPR14501[B]) antibody	Abcam	Cat# ab187162
TGN46 antibody	AbD serotech/BioRad	Cat# AHP500GT
Giantin antibody	Covance	Cat# PRB-114C
GM130 antibody	SantaCruz Biotech	Cat# SC-16268
Clathrin antibody	Abcam	Cat# ab21679
HRP-conjugated goat anti-mouse antibody	Jackson ImmunoResearch	Cat# 115-035-003
HRP-conjugated goat anti-rabbit antibody	Jackson ImmunoResearch	Cat# 111-035-144

**Table 3.** Bacterial and virus strains

	Source	Identifier
E.coli DH5alpha	Thermo Fisher Scientific	Cat# 18265017
E.coli Stbl3	Invitrogen	Cat# C737303
E.coli DB3.1	Invitrogen	Cat# 11782-018
E.coli BL21(DE3)	New England Biolabs	Cat# C2527H

**Table 4.** Chemicals, peptides, and recombinant proteins

	Source	Identifier
X-tremeGENE 9 DNA Transfection Reagent	Merck	Cat# 6365809001
ANTI-FLAG M2 Affinity gel	Merck	Cat# A2220-5ML
Polybrene	Santa Cruz Biotechnology	Cat# sc-134220
LysoTracker Deep Red	Invitrogen	Cat# L12492
Human Transferrin AlexaFluor647	Jackson ImmunoResearch	Cat# 009-600-050
Glutathione Sepharose 4B	Merck	Cat# GE17-0756-01
GFP-Trap agarose	Proteintech/Chromotek	Cat# gta
SUMOstar protease	LifeSensors	Cat# 4110-500UNITS
Amylose Resin	New England Biolabs	Cat# E8021L
Pierce Cell surface Biotinylation and Isolation kit	Thermo Fisher Scientific	Cat# A44390
DirectPCR lysis reagent	Nordic BioSite	Cat# 250-301-C
cOmplete, EDTA-free Protease inhibitor Cocktail	Roche	Cat# 5056489001
Supersignal west dura extended duration substrate	Thermo Fisher Scientific	Cat# 34075

**Table 5.** Experimental models: Cell lines

	Source	Identifier
HeLa T-Rex Flp-In	Gift from Anthony Thige and Stephen S. Taylor	Tighe et al. (2008)
HEK-FT	Invitrogen	R70007

**Table 6.** Recombinant DNA

Plasmid	Source	Identifier
pENTR-LYST	This paper	NM_000081
pENTR-ALFY	This paper	NM_014991
pENTR-WDFY4	This paper	NM_020945
pENTR-LRBA	This paper	NM_006726
pENTR223-NBEA	DF/HCC DNA Resource Core PlasmID Respository	PlasmID ID HsCD00399415, IMAGE:100069291
pENTR-NBEAL1	This paper	NM_001114132
pENTR-NBEAL2	This paper	NM_015175
pENTR-NSMAF	This paper	NM_003580
pDestFlpIn-3xFlag-EGFP-LYST	This paper	
pDestFlpIn-tdNGFlag-LYST	This paper	
pDestFlpIn-3xFlag-EGFP-ALFY	This paper	
pDestFlpIn-3xFlag-EGFP-WDFY4	This paper	
pDestFlpIn-3xFlag-EGFP-LRBA	This paper	
pDestFlpIn-3xFlag-EGFP-NBEA	This paper	
pDestFlpIn-3xFlag-EGFP-NBEAL1	This paper	
pDestFlpIn-3xFlag-EGFP-NBEAL2	This paper	
pDestFlpIn-3xFlag-EGFP-NSMAF	This paper	
pcDNA5-FRT-TO-WDR81-EGFP-3xFlag	This paper	NM_001163809
pDestFlpIn-3xFlag-EGFP-SUMOStar-LRBA	This paper	
pDestFlpIn-3xFlag-EGFP-SUMOStar-NBEAL2	This paper	
pDestFlpIn-3xFlag-EGFP-NBEAL2(1-1792)	This paper	
pDestFlpIn-3xFlag-EGFP-NBEAL2(1792-2754)	This paper	
pLVX-SV40-mScarlet-I-RAB1A	This paper	
pLVX-SV40-mScarlet-I-RAB2A	This paper	
pLVX-SV40-mScarlet-I-RAB3A	This paper	
pLVX-SV40-mScarlet-I-RAB4A	This paper	
pLVX-SV40-mScarlet-I-RAB5A	This paper	
pLVX-SV40-mScarlet-I-RAB6A	This paper	
pLVX-SV40-mScarlet-I-RAB7A	This paper	
pLVX-SV40-mScarlet-I-RAB8A	This paper	
pLVX-SV40-mScarlet-I-RAB9A	This paper	
pLVX-SV40-mScarlet-I-RAB10	This paper	
pLVX-SV40-mScarlet-I-RAB11A	This paper	
pLVX-SV40-mScarlet-I-RAB12	This paper	
pLVX-SV40-mScarlet-I-RAB13	This paper	
pLVX-SV40-mScarlet-I-RAB14	This paper	
pLVX-SV40-mScarlet-I-RAB15	This paper	
pLVX-SV40-mScarlet-I-RAB17	This paper	
pLVX-SV40-mScarlet-I-RAB18	This paper	
pLVX-SV40-mScarlet-I-RAB19	This paper	
pLVX-SV40-mScarlet-I-RAB20	This paper	
pLVX-SV40-mScarlet-I-RAB21	This paper	
pLVX-SV40-mScarlet-I-RAB22A	This paper	
pLVX-SV40-mScarlet-I-RAB23	This paper	
pLVX-SV40-mScarlet-I-RAB24	This paper	
pLVX-SV40-mScarlet-I-RAB25	This paper	
pLVX-SV40-mScarlet-I-RAB26	This paper	
pLVX-SV40-mScarlet-I-RAB27A	This paper	
pLVX-SV40-mScarlet-I-RAB28	This paper	
pLVX-SV40-mScarlet-I-RAB29	This paper	
pLVX-SV40-mScarlet-I-RAB30	This paper	
pLVX-SV40-mScarlet-I-RAB31	This paper	
pLVX-SV40-mScarlet-I-RAB32	This paper	
pLVX-SV40-mScarlet-I-RAB33B	This paper	
pLVX-SV40-mScarlet-I-RAB34	This paper	
pLVX-SV40-mScarlet-I-RAB35	This paper	
pLVX-SV40-mScarlet-I-RAB36	This paper	
pLVX-SV40-mScarlet-I-RAB37	This paper	
pLVX-SV40-mScarlet-I-RAB38	This paper	
pLVX-SV40-mScarlet-I-RAB39A	This paper	
pLVX-SV40-mScarlet-I-RAB40A	This paper	
pLVX-SV40-mScarlet-I-RAB41	This paper	
pLVX-SV40-mScarlet-I-RAB42	This paper	
pLVX-SV40-mScarlet-I-RAB43	This paper	
pLVX-SV40-ARF1-mScarlet-I	This paper	
pLVX-SV40-ARF3-mScarlet-I	This paper	
pLVX-SV40-ARF4-mScarlet-I	This paper	
pLVX-SV40-ARF5-mScarlet-I	This paper	
pLVX-SV40-ARF6-mScarlet-I	This paper	
pLVX-SV40-SAR1A-mScarlet-I	This paper	
pLVX-SV40-mScarlet-I-COPE	This paper	
pSBbi-pur-mScarlet-I-SEC31A	This paper	
pLVX-SV40-AP1M1-mScarlet-I	This paper	
pLVX-SV40-AP2M1-mScarlet-I	This paper	
pLVX-SV40-AP3M1-mScarlet-I	This paper	
pLVX-SV40-AP4M1-mScarlet-I	This paper	
pLVX-SV40-GGA1-mScarlet-I	This paper	
pLVX-SV40-GGA2-mScarlet-I	This paper	
pLVX-SV40-GGA3-mScarlet-I	This paper	
pLVX-SV40-CLINT1-mScarlet-I	This paper	
pSBbi-pur-mScarlet-I-CLTC	This paper	
pSBbi-pur-mScarlet-I-LRBA	This paper	
pSBbi-pur-mScarlet-I-NBEA	This paper	
Str-li-CTLA4-SBP-EGFP	This paper	
Str-li-CTLA4-SBP-mScarlet-I	This paper	
Str-li-CTLA4-SBP-mTagBFP2	This paper	
pGEX-5X3	GE Healthcare	Cat# 27-4586-01
pDest15-CTLA4(187–223)	This paper	
pDest15-CTLA4(187–202)	This paper	
pDest15-CTLA4(194–209)	This paper	
pDest15-CTLA4(201–215)	This paper	
pDest15-CTLA4(208–223)	This paper	
pDest15-CTLA4(201–215)Y201A	This paper	
pDest15-CTLA4(201–215)V202A	This paper	
pDest15-CTLA4(201–215)K203A	This paper	
pDest15-CTLA4(201–215)M204A	This paper	
pDest15-CTLA4(201–215)P205A	This paper	
pDest15-CTLA4(201–215)P206A	This paper	
pDest15-CTLA4(201–215)T207A	This paper	
pDest15-CTLA4(201–215)E208A	This paper	
pDest15-CTLA4(201–215)P209A	This paper	
pDest15-CTLA4(201–215)E210A	This paper	
pDest15-CTLA4(201–215)C211A	This paper	
pDest15-CTLA4(201–215)E212A	This paper	
pDest15-CTLA4(201–215)K213A	This paper	
pDest15-CTLA4(201–215)Q214A	This paper	
pDest15-CTLA4(201–215)F215A	This paper	
pDest15-EPHA7(579–998)	This paper	
pDest15-EFNB2(251–333)	This paper	
pDest15-ROBO2(883–1378)	This paper	
pDest15-FGFR4(388–802)	This paper	
pDest15-FLRT2(563–660)	This paper	
pDest15-JAG1(1093–1218)	This paper	
pDest15-TGFBR3(810–851)	This paper	
pDest15-SDK1(2031–2213)	This paper	
pSpCas9(BB)-2A-Puro (PX459) V2.0	Addgene	Cat#62988
pX459-ALFYg1	Søreng et al. (2022)	
pX459-ALFYg2	Søreng et al. (2022)	
pX459-LRBAg1	This paper	
pX459-LRBAg2	This paper	
pX459-NBEAg1	This paper	
pX459-NBEAg2	This paper	
pX459-NBEAL1g1	This paper	
pX459-NBEAL1g2	This paper	
pX459-NBEAL2g1	This paper	
pX459-NBEAL2g2	This paper	
pCMV-VSV-G	Addgene	Cat#8454
psPAX2	Addgene	Cat#12260
pOG44	Invitrogen	Cat#V600520
SB100X	Addgene	Cat# 127909
pSBbi-pur	Addgene	Cat# 60523
GFP-CHC17KDP	Addgene	Cat# 59799
Str-Ii_VSVG-SBP-mCherry	Addgene	Cat# 65301
pDest15	Invitrogen	Cat# 11802014
pEGFP-N1-hFGFR4	Haugsten et al. (2014)	

Plasmids and stable cell lines generated in this study are available from the lead contact.

### Cell culture

HeLa T-Rex Flp-In and HEK-FT cells were grown and maintained in Dulbecco’s modified Eagle’s medium supplemented with 10% FBS, 5 U/ml penicillin, and 50 µg/ml streptomycin at 37°C, 5% CO_2_. Media for original HeLa T-Rex Flp-In cells was also supplemented with 5 µg/ml blasticidin and 100 µg/ml zeocin to maintain the TET-repressor and the FRT site. Media for HeLa T-Rex Flp-In cells with a stably integrated gene of interest in the FRT site was supplemented with 5 µg/ml blasticidin and 100 µg/ml hygromycin B. Gene expression was induced by 24–48 h treatment with 1 µg/ml doxycycline.

### Plasmids and cloning

Human BDCPs were amplified by PCR from human cDNA libraries from U2OS cells (for LYST, ALFY, LRBA, NBEAL1, NBEAL2, WDR81, and NSMAF) or primary B lymphocytes (kind gift from Karin M. Gilljam, for WDFY4) as 3–4 kb fragments and cloned into pENTR1A/2B/3C or pcDNA5 FRT/TO backbones by Gibson assembly. Flp-In compatible expression plasmids containing 3xFlag-EGFP fusions of BDCPs under the control of a tet-on CMV promoter were generated by Gateway recombination cloning. Sleeping Beauty transposition compatible vectors of LRBA and NBEA were generated by Gibson assembly of corresponding BDCP and mScarlet into pSBbi-pur backbone. Lentiviral transfer plasmids for RAB small GTPases were generated by PCR amplification from human cDNA libraries or gene synthesis (for RAB37, RAB39A, and RAB41) (Thermo Fisher Scientific) followed by restriction enzyme cloning into pLVX-SV40-mScarlet backbone to generate mScarlet-Rabs fusions under control of SV40 promoter. Lentiviral transfer plasmids for Arf and Sar1A small GTPAses, COPE, AP1M1, AP2M1, AP3M1, AP4M1, GGA1, GGA2, GGA3, and CLINT1 were generated by PCR amplification of corresponding genes from human cDNA library from U2OS cells followed by Gibson assembly together with mScarlet into pLVX-SV40 backbone to generate N-terminal mScarlet fusion (for COPE) or C-terminal mScarlet fusions (for other proteins) under SV40 promoter. Sleeping Beauty transposition compatible vectors of SEC31A and clathrin heavy chain were generated by PCR amplification of SEC31A from human cDNA library or clathrin heavy chain from GFP-CHC17KDP vector (Addgene plasmid 59799, gift from Stephen Royle), followed by Gibson assembly together with mScarlet into pSBbi-pur backbone. RUSH-compatible plasmids of CTLA4 were generated by PCR amplification of CTLA4 from the human cDNA library followed by Gibson assembly instead of VSVG into Str-Ii_VSVG-SBP-mCherry vector. Vectors for bacterial expression of the full-length cytosolic tail of CTLA4 or its deletion or point mutants were generated by PCR amplification of CTLA4(187–223) from human cDNA library or DNA synthesis for CTLA4(187–202), CTLA4(194–209), CTLA4(201–215), and CTLA4(208–223) followed by restriction enzyme cloning into pENTR1A/2B/3C vectors and Gateway recombination cloning into pDest15 vector. Vectors for bacterial expression of the cytosolic tails of EPHA7, EFNB2, ROBO2, FGFR4, FLRT2, JAG1, TGFBR3, and SDK1 were generated by PCR amplification from the human cDNA library, followed by restriction enzyme cloning into pENTR1A/2B/3C and Gateway recombination cloning into pDest15 vector. To generate vectors for CRISPR KO of BDCPs oligonucleotide guides to target LRBA (guide 1 5′-GTG​AGA​GTA​TTA​ACT​GCA​TGG-3′ or guide 2 5′-GCA​TCA​GGA​CCA​TAC​TTC​TG-3′), NBEA (guide1 5′-GCA​GCT​ACA​GCA​TCA​CTG​TCA-3′ or guide 2 5′-GGT​TGG​TGG​AGA​ATT​TGA​CT-3′), NBEAL1 (guide 1 5′-GGA​AGC​AGA​GAT​ATT​CCT​GG-3′ or guide 2 5′-GAG​ATT​ACC​TGA​AGC​TGT​GGT-3′) or NBEAL2 (guide 1 5′-GAG​CAC​ATG​CAG​CTC​ATC​CAG-3′ or guide 2 5′-GAC​CTG​GGT​TAC​CTG​CAG​CAG-3′) were cloned into BbsI sites of pSpCas9(BB)-2A-Puro (PX459) V2.0 plasmid.

### Production of lentiviral particles and transduction of HeLa cells

10^6^ HEK-FT cells were plated in a 10-cm dish and transfected the next day with 1.6 µg of each of pCMV-VSV-G, psPAX2, and transfer plasmids. The medium was changed after 24 h and lentivirus-containing medium was collected and filtered through Acrodisc 0.45 µm Supor membrane syringe filter at 48 and 72 h after transfection. For cell infection, 10^5^ HeLa cells were plated in wells of a six-well plate and the next day the cell medium was exchanged for 2 ml fresh media with 0.5 ml of lentivirus-containing medium and 8 µg/ml of polybrene (Santa Cruz Biotechnology). The medium was changed 24 h after infection for fresh medium containing 2 µg/ml of puromycin. A pool of puromycin-selected cells was used for experiments.

### Generation of stably transfected cell lines

The stably transfected cell lines used in this paper were generated using either Flp-FRT recombination, the Sleeping Beauty transposon system, or by infection with lentiviral particles. For the Flp-FRT recombination approach, 10^5^ HeLa T-Rex Flp-In cells were plated in wells of a 6-well plate and 24 h later co-transfected with pOG44 Flp-recombinase expression vector and one of the pDestFlpIn or pcDNA5-FRT-TO vectors in a ratio 10:1 using X-tremeGENE 9 DNA transfection reagent. 24 h after transfection, the cells were trypsinized and plated into 10-cm dishes, followed by medium change for complete growth media containing 200 µg/ml Hygromycin B 24 h later. Individual clones resistant to Hygromycin B were picked 2 wk after initiation of selection and tested by western blotting and confocal imaging for expression of the protein of interest. To generate stably transfected cells using the Sleeping Beauty transposon system approach, 10^5^ HeLa T-Rex Flp-In cells were plated in wells of a six-well plate and 24 h later co-transfected with SB100x Sleeping Beauty transposase expression vector and one of pSBbi-pur vectors in 1:1 ratio using X-tremeGENE 9 DNA transfection reagent. 24 h after transfection cells were trypsinized and plated into 10-cm dishes, followed by medium change for complete growth media containing 2 µg/ml of puromycin. The pool of puromycin-selected cells was used for experiments. The generation of stably transfected cells with the lentiviral approach is described in the Virus production and transduction section.

### Generation of BDCPs KO cell line by CRISPR/Cas9

HeLa T-Rex Flp-In cell lines lacking the reference isoforms of ALFY, LRBA, NBEA, NBEAL1, and NBEAL2 were generated by sequential transfection of HeLa T-Rex Flp-In wt cells with pX459 plasmid encoding SpCas9 and guide RNAs targeting NBEAL1, NBEA, LRBA, ALFY, and NBEAL2, as described in the Plasmids and cloning section. Transfected cells were selected by culturing for 2 days in complete media with 2 µg/ml of puromicin, followed by plating 10^3^ cells in a 15-cm dish for clonal growth. Single clones were isolated 2 wk after plating and tested for indel mutations by genotyping (for NBEAL1) or western blotting (for ALFY, LRBA, NBEA, and NBEAL2). For genotyping, total genomic DNA was isolated from each clone using DirectPCR Lysis Reagent and genomic regions targeted by gRNAs were amplified using primer pairs 5′-ACA​TCT​GTG​TTG​CAG​ACG​AG-3′ and 5′-AGC​TCC​ATA​TCA​ACT​ACA​AAC​ATC-3′ for guide 5′-GGA​AGC​AGA​GAT​ATT​CCT​GG-3′ or primer pairs 5′-CAG​GGA​TAT​GGG​AGG​GCA​AAT-3′ and 5′-CAG​AGT​TTT​CTA​GAA​GCC​AGG​TC-3′ for guide 5′-GAG​ATT​ACC​TGA​AGC​TGT​GGT-3′. The obtained PCR products were sequenced using primers 5′-GTT​GCA​GAC​GAG​AAG​GCA​C-3′ and 5′-TGT​ATG​CAG​AAT​GTG​CAG​G-3′ for guide 5′-GGA​AGC​AGA​GAT​ATT​CCT​GG-3′ or primers 5′-TTT​GAG​TGA​CAT​GAG​AAG​CC-3′ and 5′-CAT​CAA​TTA​GCT​ATG​TCT​CTA​TGA​G-3′ for guide 5′-GGA​AGC​AGA​GAT​ATT​CCT​GG-3′.

### Western blotting

For western blot analysis, cells grown to confluency were harvested in lysis buffer (50 mM Tris-HCl pH 7.4, 150 mM NaCl, and 1% Triton X-100) supplemented with complete protease inhibitor cocktail (05056489001; Roche) for 10 min on ice. The lysates were centrifuged at 5,000 × *g* for 10 min at 4°C to pellet cell debris. The supernatants were separated by SDS-PAGE and transferred to a nitrocellulose membrane (Amersham Protan Premium 0.45 µm; Cytiva). The membrane was blocked with PBS supplemented with 0.1% Tween and 5% skim milk powder for 1 h at room temperature and incubated with primary antibodies overnight at 4°C, followed by incubation with an anti-rabbit or anti-mouse HRP-conjugated secondary antibody in PBS + 0.1% Tween. The HRP signal was visualized by incubation of membranes with Supersignal West Dura Extended duration substrate (34075; Thermo Fisher Scientific) for 5 min followed by imaging with the Chemidoc MP Imaging system (BioRad).

### Recombinant protein production and GST-Pulldown assay

GST and GST-fusion proteins or MBP and MPB-fusion proteins were expressed in BL21(DE3) *E.coli* strain and purified by immobilization on Glutathione Sepharose 4B beads (GST) or Amylose resin. Briefly, plasmids were transformed into BL21(DE3) *E. coli* strain and grown as overnight cultures in ∼5 ml of LB media at 37°C with 220 RPM shaking. Overnight cultures were used to inoculate ∼100 ml LB cultures and grown at 37°C until OD600 reached 0.7, then induced with 0.5 mM IPTG and incubated for 4 h at 20°C with 220 RPM shaking. Bacterial cultures were then pelleted at 12,000 × *g* for 10 min, pellets suspended and sonicated in lysis buffer (50 mM Tris-HCl pH 7.4, 150 mM NaCl, and 1% Triton X-100) and clarified by centrifugation at 12,000 × *g* for 10 min at 4°C. The obtained supernatants were incubated for 2 h with 150 μl of packed glutathione sepharose 4B resin (for GST-fusion proteins) or amylose resin (for MBP-fusion proteins) followed by washing of beads with immobilized proteins with the lysis buffer. MBP-fusion proteins were eluted with 20 mM maltose in the lysis buffer. For GST-pulldown assays, glutathione sepharose beads with equal amounts of immobilized proteins were incubated overnight at 4°C with either 500 μl of HeLa cells lysates expressing 3xFlag-EGFP-fusion of BDCPs or 1 µg of MBP-fusion proteins or 1 µg of NBEAL2 or 0.2 µg of LRBA. The beads were then washed six times with lysis buffer (50 mM Tris-HCl pH 7.4, 150 mM NaCl, and 1% Triton X-100) and the bound proteins were eluted by boiling for 5 min in 2x SDS gel loading buffer (100 mM Tris pH 6.8, 4% SDS, 20% Glycerol, 0.2% Bromphenol blue, and 200 mM DTT).

Full-length NBEAL2 or LRBA were affinity purified on GFP-Trap agarose beads from cell lysates of FreeStyle 293-F cells transiently transfected with 3xFlag-EGFP-SUMOstar-NBEAL2 or 3xFlag-EGFP-SUMOstar-LRBA and eluted with SUMOstar protease, resulting in untagged NBEAL2 and LRBA proteins that were used for GST-pulldown assays.

### Live cell imaging

Cells grown in eight-well Lab-Tek II chambered coverglass were imaged live in FluoreBrite DMEM medium (Gibco) on Andor Dragonfly 505 high-speed confocal platform equipped with Okolab cell incubator with temperature, CO_2_, and humidity control (at 37°C and 5% CO_2_) using NIKON Apo TIRF 60×/1.49 NA or NIKON Apo 100x/1.45 NA oil immersion objectives and Zyla4.2 sCMOS 2,048 × 2,048 camera. The spinning disc confocal mode was used for all figures. Between 30 and 60 frames at one to two frames per second were acquired for each field of view. Two proteins were considered colocalized when signals from both proteins co-migrated within the punctuated or tubular structure during the timeframe of the experiment.

For the transferrin internalization experiment, 200 μl of FluoreBrite DMEM media containing 20 µg/ml of AlexaFluor647 conjugated human transferrin was added to the well of Lab-Tek II chambered coverglass with cells grown in 200 μl of FluoreBrite DMEM media to obtain a final transferrin concentration of 10 µg/ml. The imaging of the cells was started within 30 s after the addition of transferrin.

### Immunofluorescent staining of fixed cells

Cells grown in 8-well µ-Slide chambered coverglass (Ibidi) were fixed at 37°C for 30 min by adding an equal volume of solution of 8% PFA and prewarmed to 37°C directly to culture media. After fixation cells were washed, permeabilized with 0.05% saponin, and stained with antibodies against LRBA (HPA023597, 1:500), TGN46 (AHP500GT, 1:500), Giantin (PRB-114C, 1:1,000), GM130 (SC-16268, 1:200), or clathrin (ab21679, 1:200). Cells were imaged on Nikon CrestOptics X-Light V3spinning disc confocalsystem (Kinetix-M-C sCMOS 3,200 × 3,200 camera) or Nikon CSU-W1 SoRa superresolution systems (Prime BSI sCMOS 2,048 × 2,048 camera) using NIKON Apo 100x/1.45 NA oil immersion objective.

### Image analyses

Quantification of colocalization between AP1M1 and LRBA or NBEA was performed using manual segmentation of fluorescent signal from corresponding proteins with LABKIT labeling and segmentation plugin (Arzt et al., 2022) in Fiji/ImageJ (Schindelin et al., 2012). AP1M1-positive objects located within the diameter of LRBA- or NBEA-positive objects were scored as colocalized. The ratio of AP1M1-positive objects colocalizing with LRBA or NBEA to the total number of AP1M1 objects per cell (150–300 objects per cell, 15–20 cells per condition) is presented as separate dots in a boxplot in Fig. 4 B. Quantification of colocalization between transferrin or ARF1 and BDCPs was performed by automatic segmentation of fluorescent signal from corresponding proteins with NIS-Elements AR analysis software v5.30 (Nikon Instruments). The ratio of segmented BDCP-positive objects overlapping with ARF1- or transferrin-positive objects to the total number of BDCP-positive objects per cell (15–20 cells for colocalization with ARF1 and 50–100 cells for colocalization with transferrin) is presented as separate dots in boxplots in Fig. 3 E and Fig. 5 G.

Densitometric quantification of Western blots was performed using ImageJ. A rectangular ROI sized to enclose the band with the largest area was created and the same ROI was used to measure the background-substracted integrated density of the rest of the bands. The densitometric values were normalized to the intensity of actin loading control for Fig. 10 C.

### Retention using selective hook (RUSH) assay

HeLa cells were plated in eight-well Lab-Tek II chambered coverglass and transfected with Str-li-CTLA4-SBP-EGFP/-mScarlet or -mTagBFP plasmids. Transfected cells were imaged 24 h later. The release of CTLA4 from ER was initiated by adding 200 μl of FluoreBrite DMEM media with 80 µM biotin to the well containing transfected HeLa cells in 200 μl of FluoreBrite DMEM. The imaging was started within 30 s after addition of biotin.

### Cell surface protein biotinylation and isolation

Isolation of the cell surface proteome from wild type or BDCPs KO HeLa cells was performed using Pierce Cell Surface Protein Biotinylation and Isolation kit (Thermo Fisher Scientific) according to the manufacturer’s instructions. Briefly, 10^7^ of either HeLa wt or BDCPs PKO cells were plated in 15-cm dishes. Next day, the cell media was removed and the cells were washed quickly one time with 20 ml of ambient PBS and incubated for 10 min at room temperature with 250 µg/ml solution of EZ-Link Sulfo-NHS-SS-Biotin in PBS. After incubation, the cells were washed twice with 20 ml ice-cold TBS, then scraped into 10 ml of ice-cold TBS, and pelleted at 500 × *g* for 3 min at 4°C. Cell pellets were lysed for 30 min on ice in 500 μl of lysis buffer and cell lysate was clarified by centrifugation at 15,000 × *g* for 5 min at 4°C. Clarified lysate was incubated with 250 μl of NeutrAvidin agarose slurry for 30 min at room temperature followed by washing of beads with bound proteins four times with 500 μl of wash buffer.

### On-beads protein digestion

100 μl 50 mM NH_4_HCO_3_ was added to the bead samples followed by quick vortexing. Proteins were reduced (10 mM DTT) for 20 min at 56°C and alkylated (20 mM Iodoacetamide) in the dark for 30 min at RT before digestion with 1 µg trypsin (Porcine; Promega) overnight at 37°C. The samples were centrifuged at 14,000 × *g* for 10 min to pellet the beads. The supernatant containing the digested proteins was captured, concentrated, and desalted using C18 stage tips (Affinisep).

### LC-MS/MS analysis

Samples were analyzed by a nanoElute UHPLC coupled to a timsTOFfleX mass spectrometer (Bruker Daltonics) via a CaptiveSpray ion source. Peptides were separated on a 25-cm reversed-phase C18 column (1.6 µm bead size, 120 Å pore size, 75 µm inner diameter; Ion Optics) with a flow rate of 0.3 μl/min and a solvent gradient from 0 to 35% B in 60 min. Solvent B was 100% acetonitrile in 0.1% formic acid and solvent A 0.1% formic acid in water. The mass spectrometer was operated in data-dependent parallel accumulation-serial fragmentation (PASEF) mode. Mass spectra for MS and MS/MS scans were recorded between m/z 100 and 1,700. Ion mobility resolution was set to 0.60–1.60 Vs/cm over a ramp time of 100 ms. Data-dependent acquisition was performed using 10 PASEF MS/MS scans per cycle with a near 100% duty cycle. A polygon filter was applied in the m/z and ion mobility space to exclude low m/z, singly charged ions from PASEF precursor selection. An active exclusion time of 0.4 min was applied to precursors that reached 20,000 intensity units. Collisional energy was ramped stepwise as a function of ion mobility.

### Protein identification and label-free quantitation

MS raw files were submitted to MaxQuant software version 2.0.1.0 for protein identification and were searched against the human SwissProt database (version 2020). Parameters were set as follows: carbamidomethylation as fixed modification; protein N-acetylation and methionine oxidation as variable modifications. First, the search error window of 20 ppm and the mains search error of 6 ppm. Trypsin without proline restriction enzyme option was used with two allowed miscleavages. Minimal unique peptides were set to 1 and the FDR allowed was 0.01 (1%) for peptide and protein identification. MaxQuant output files were loaded into the Perseus software. Identifications from potential contaminants and reversed sequences were removed and intensities were transformed to log_2_. Next, proteins identified in two out of three replicates were considered for further analysis. All zero-intensity values were replaced using noise values of the normal distribution of each sample. Protein abundances were compared using LFQ intensity values and a two-sample Student’s *T* test (permutation-based FDR correction [250 randomizations], FDR cut-off: 0.05, S0: 0.1).

### AlphaFold modeling

Protein sequences of reference isoforms of human LYST (Q99698), ALFY/WDFY3 (Q8IZQ1), WDFY4 (Q6ZS81), LRBA (P50851), NBEA (Q8NFP9), NBEAL1 (Q6ZS30), NBEAL2 (Q6ZNJ1), NSMAF (Q92636), and WDR81 (Q562E7) were used to model the structure of corresponding BDCPs using AlphaFold3 web server interface (https://alphafoldserver.com/). Best-ranked models had predicted template modeling (pTM) scores between 0.67 (LYST) and 0.8 (NBEAL1 and NBEAL2) and are presented in Fig. 1 A.

### Quantification and statistical analysis

All relevant statistical tests were carried out using Graphpad Prism software using statistical tests as described in the figure legends.

### Online supplemental material

Fig. S1 shows colocalization of LYST, NBEAL2, ALFY, NBEA, and LRBA with additional molecular markers of intracellular compartments. Fig. S2 demonstrates the dynamics of LRBA- or NBEA-positive tubules and shows their colocalization with additional molecular markers. Fig. S3 characterizes the trafficking of CTLA4-RUSH protein vs RAB1A, RAB6A, AP1M1, and AP2M1 molecular markers. Fig. S4 characterizes the trafficking of CTLA4-RUSH protein vs RAB5A and RAB11A and validates the CRISPR KO of reference isoforms of BDCPs. Video 1 shows the dynamics of NBEAL1- and RAB6A- positive compartments. Video 2 shows the dynamics of ALFY- and RAB5A-positive compartments. Video 3 shows the dynamics of ALFY- and RAB6A-positive compartments. Video 4 shows the dynamics of NBEA-positive tubules that contain AP1M1-mScarlet in “beads on a string” manner and endocytosed transferrin. Video 5 shows the dynamics of NBEAL1-positive tips of CTLA4-containing secretory tubules. Video 6 shows the formation of NBEAL1 or LRBA positive secretory carriers.

## Supplementary Material

SourceData F1 is the source file for Fig. 1.

SourceData F9 is the source file for Fig. 9.

SourceData F10 is the source file for Fig. 10.

SourceData FS4 is the source file for Fig. S4.

## Data Availability

All data are presented in the paper and Online supplemental material and are available upon request.
