# Supplementary figures and images for: BEACH domain proteins function as cargo-sorting adaptors in secretory and endocytic pathways
Source: J Cell Biol. 2024 Nov 8;223(12):e202408173. doi: 10.1083/jcb.202408173 (PMC11554844; doi:10.1083/jcb.202408173)

Figure 1B

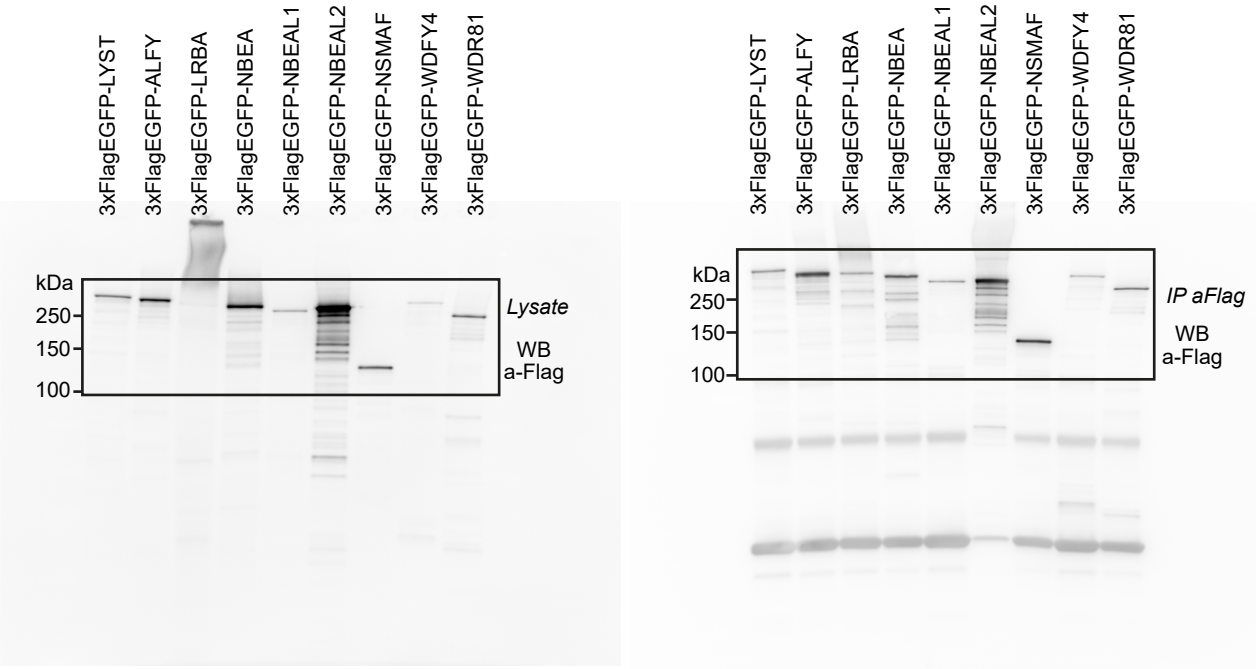

Supplement: SourceData F1 — is the source file for Fig. 1. [file JCB_202408173_SourceDataF1.pdf]

9B

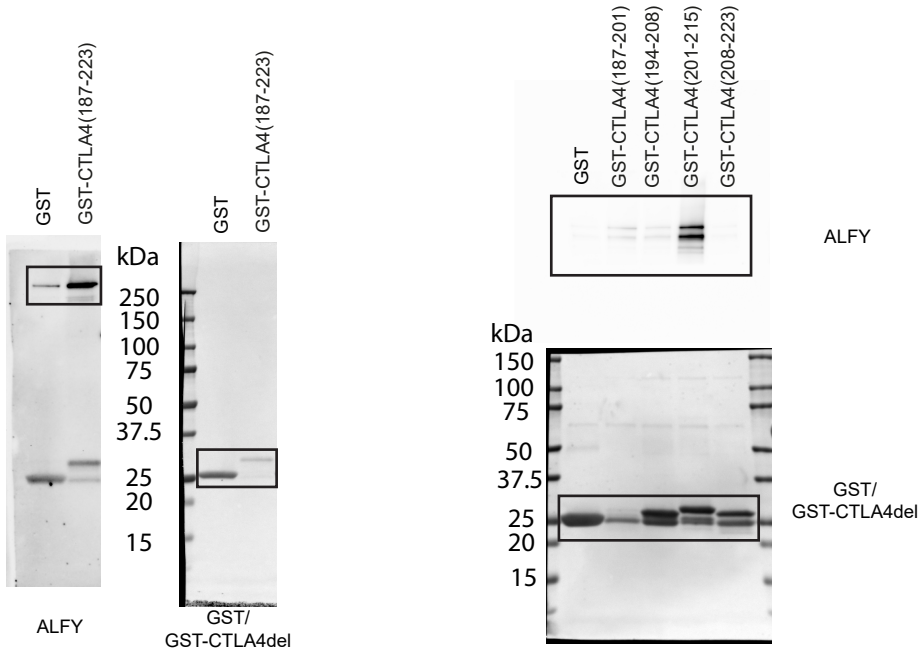

9C

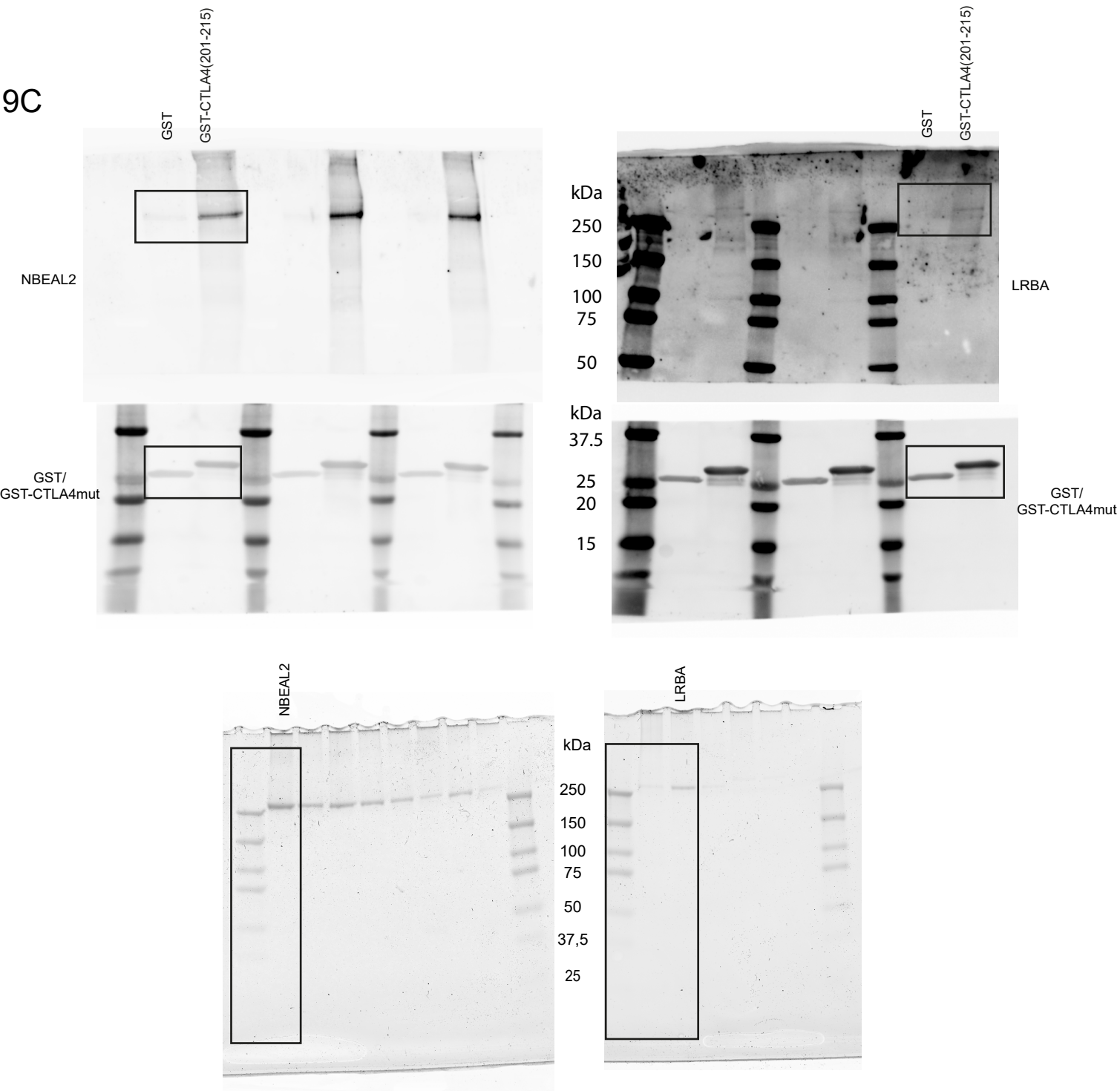

9D

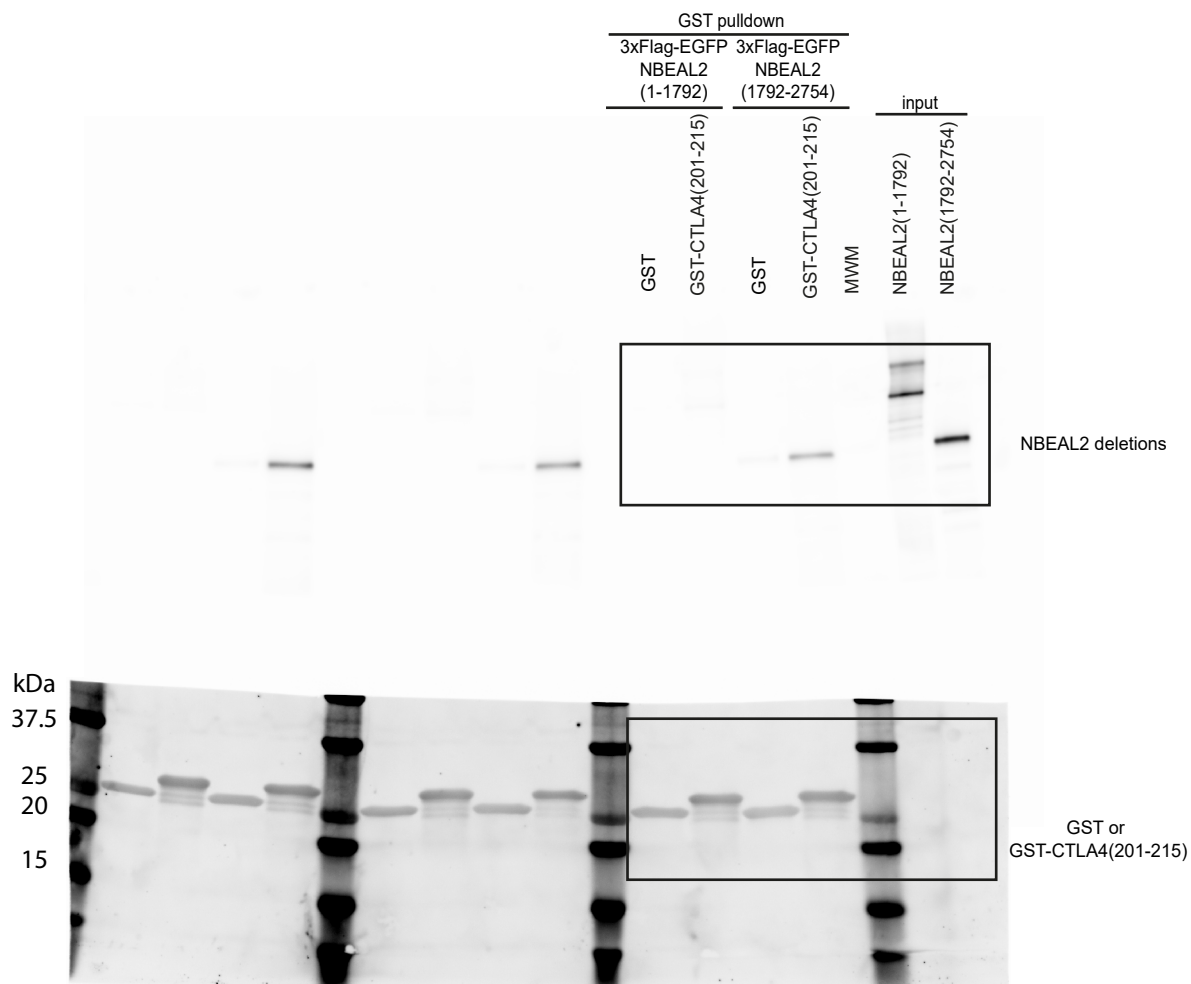

9E

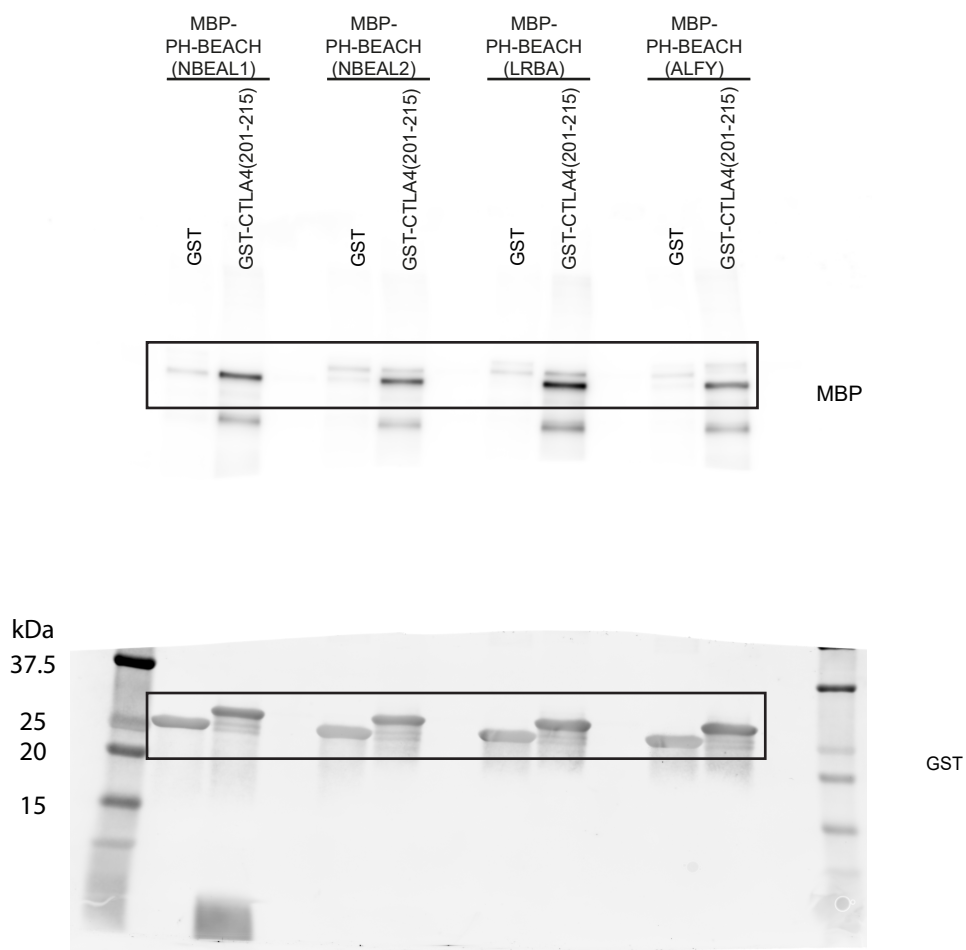

9F

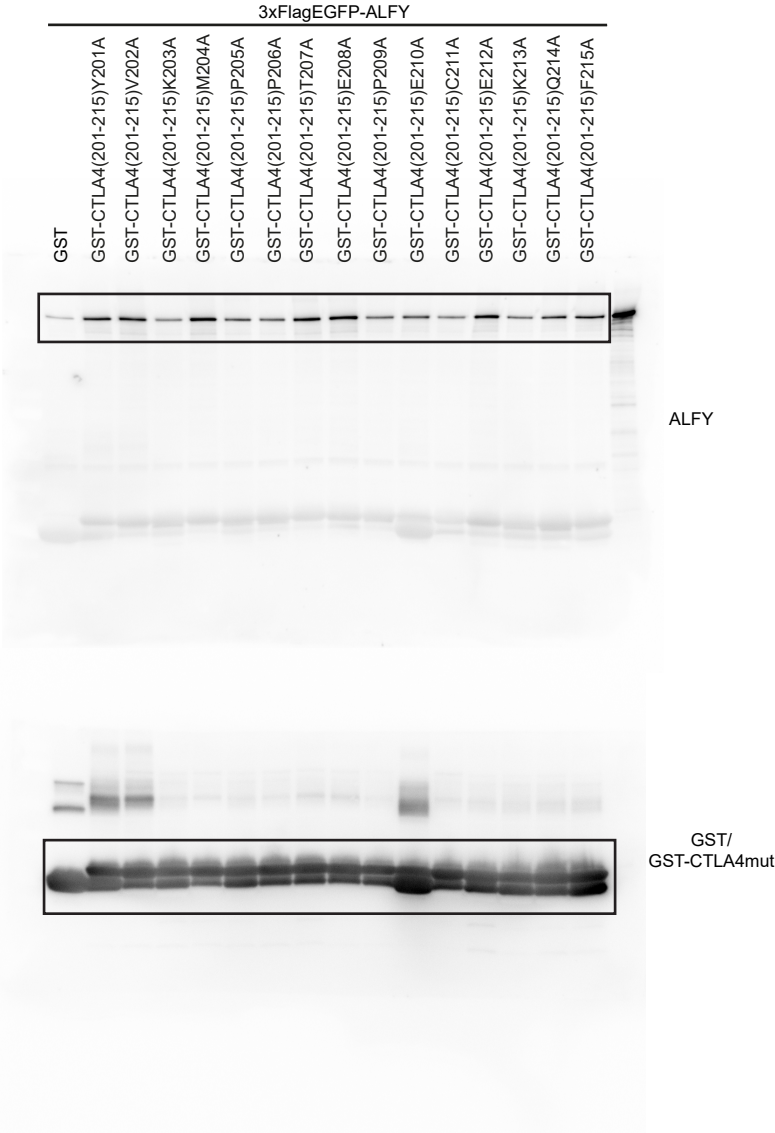

9H

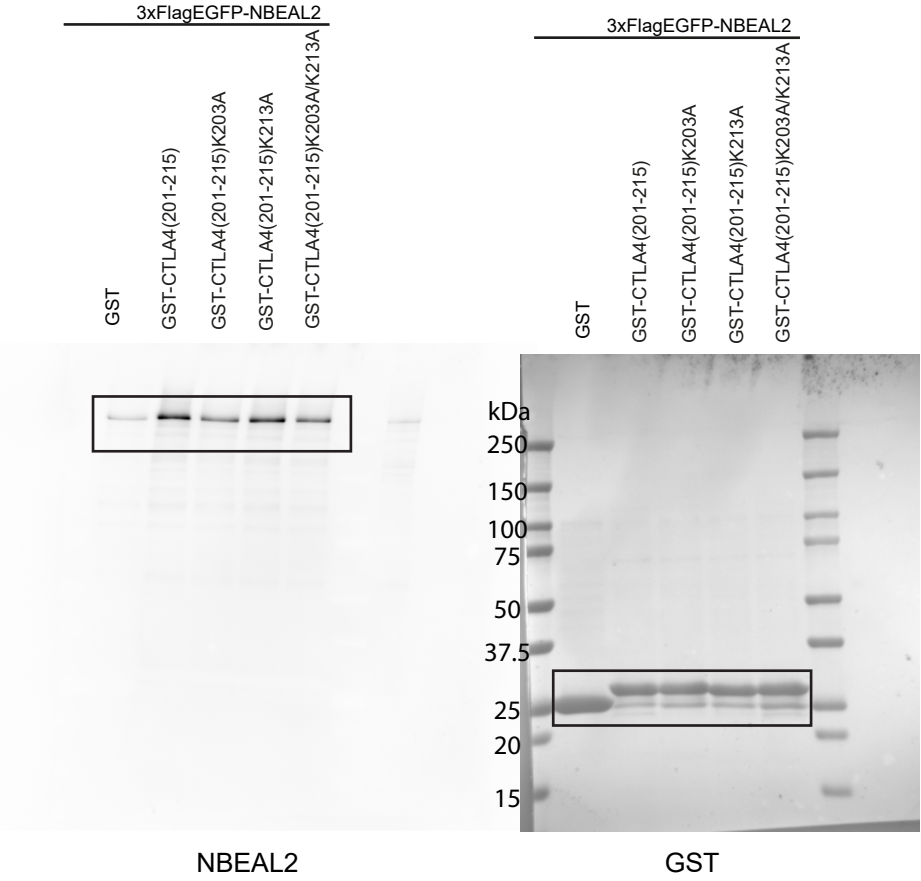

9H

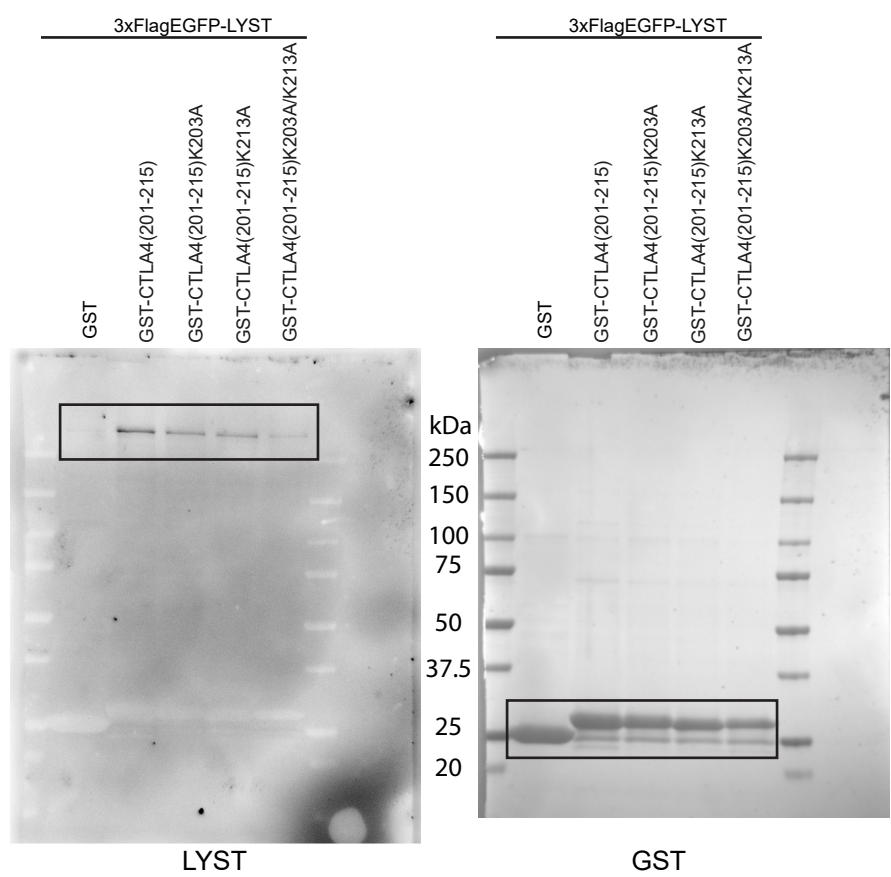

9H

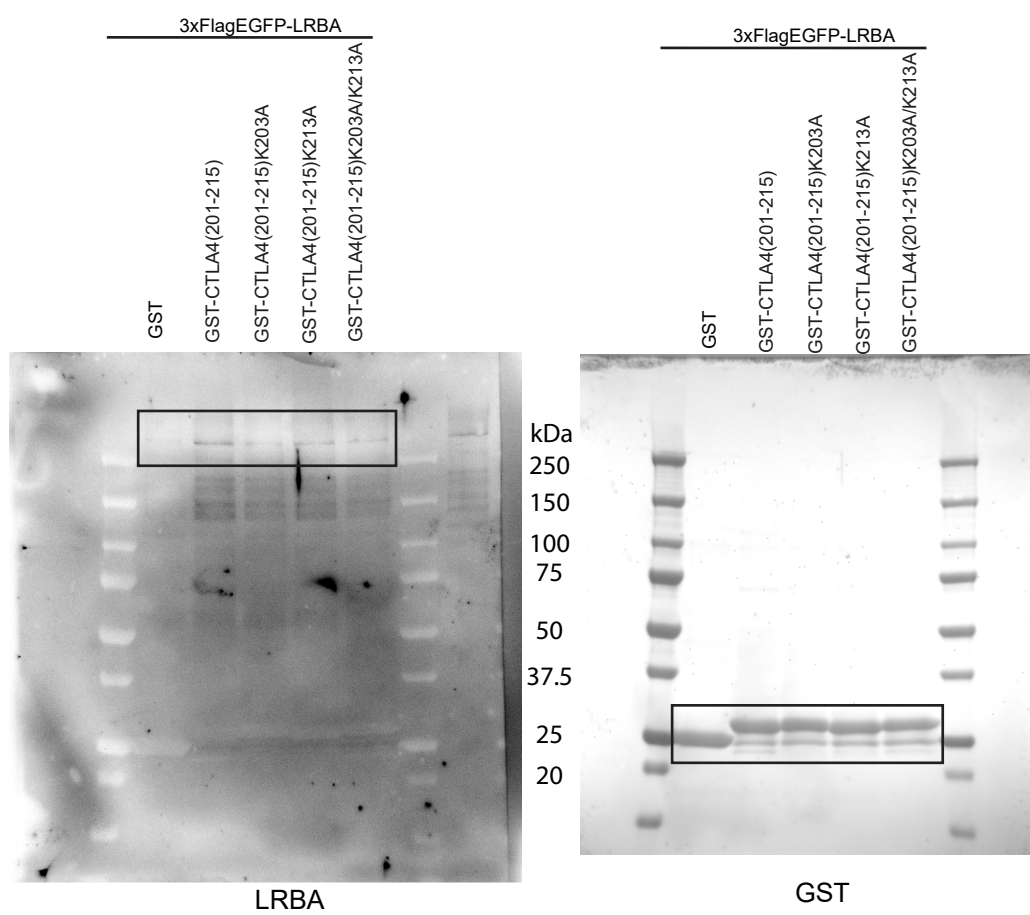

Supplement: SourceData F9 — is the source file for Fig. 9. [file JCB_202408173_SourceDataF9.pdf]

10A

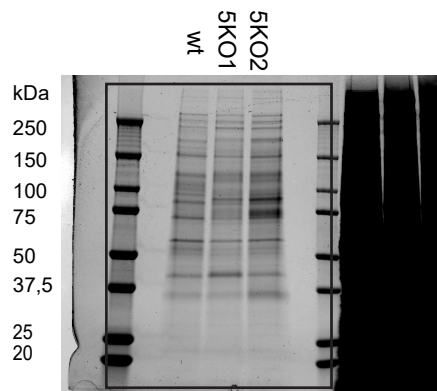

10C

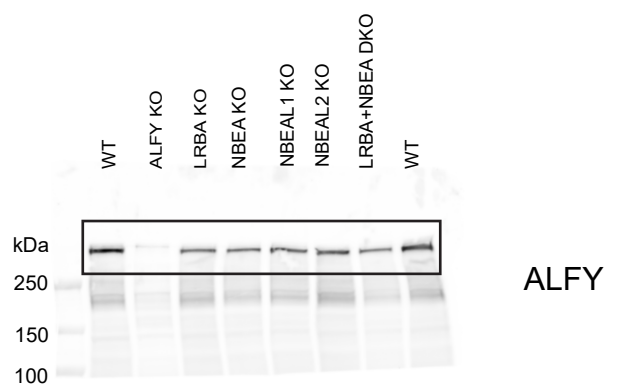

10B

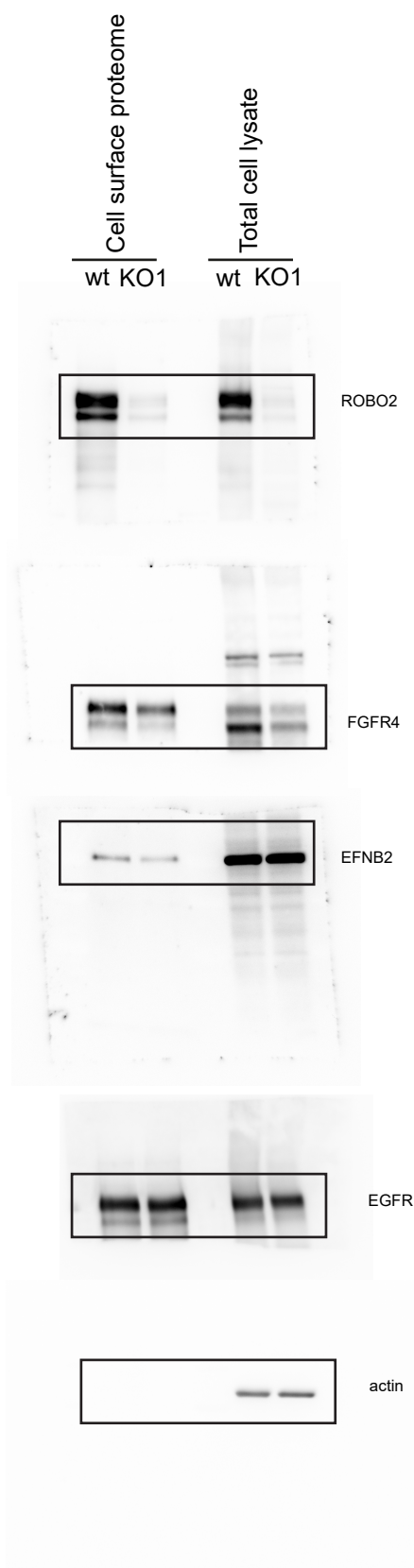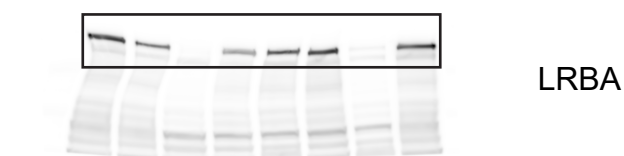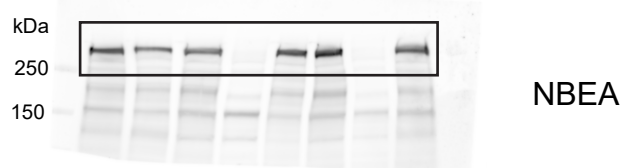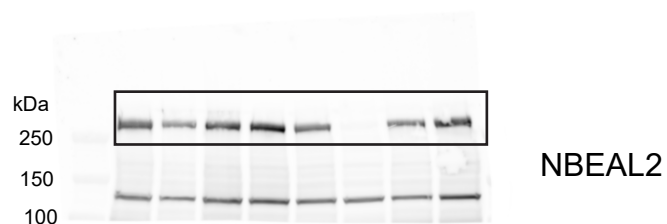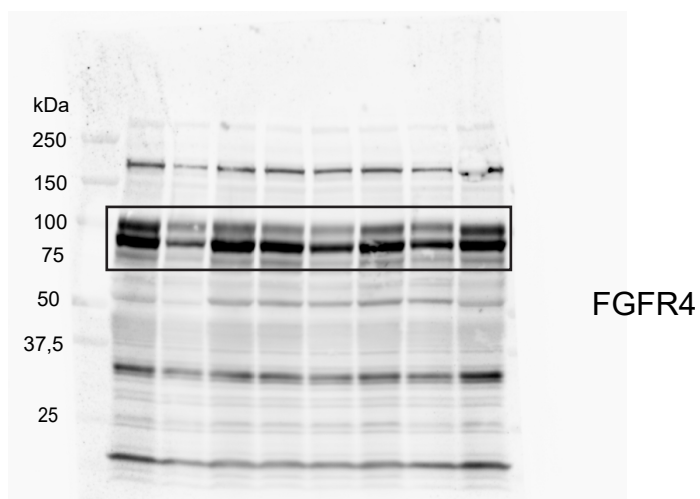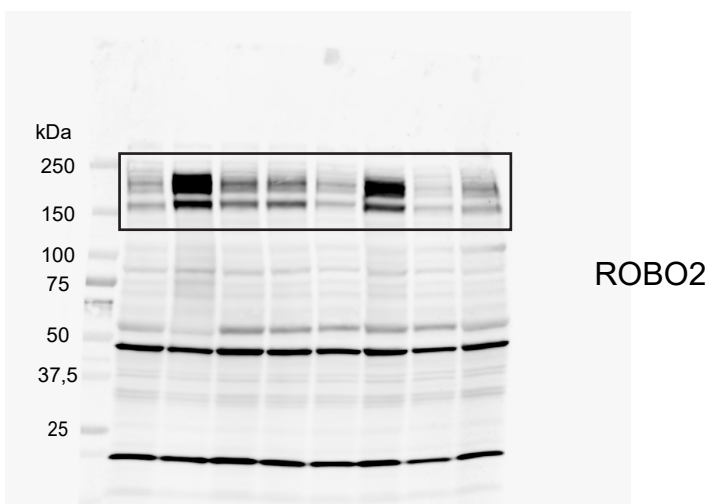

10C

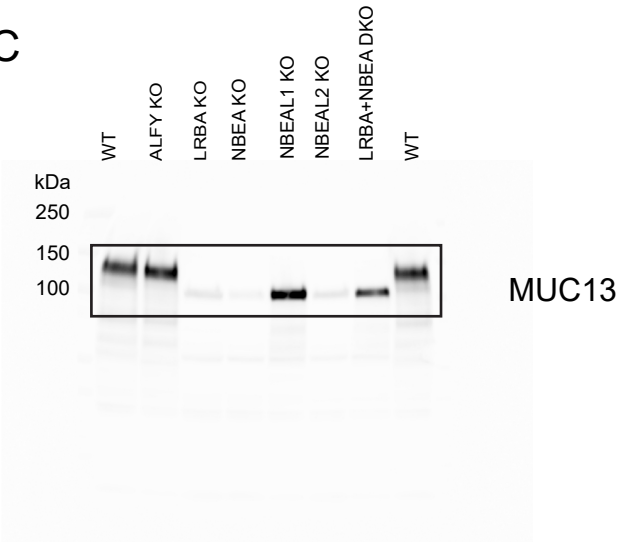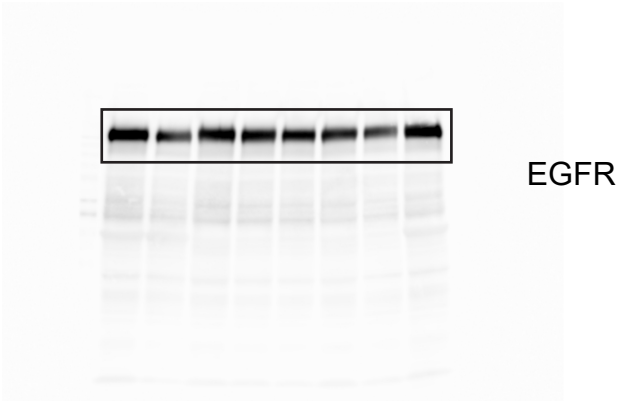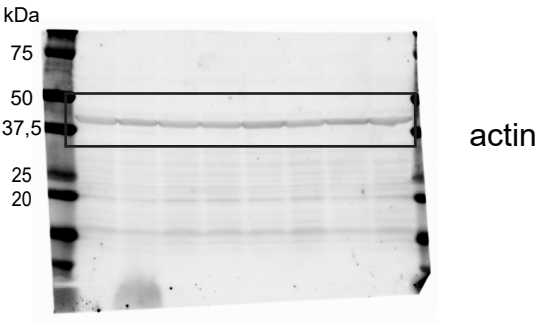

10E

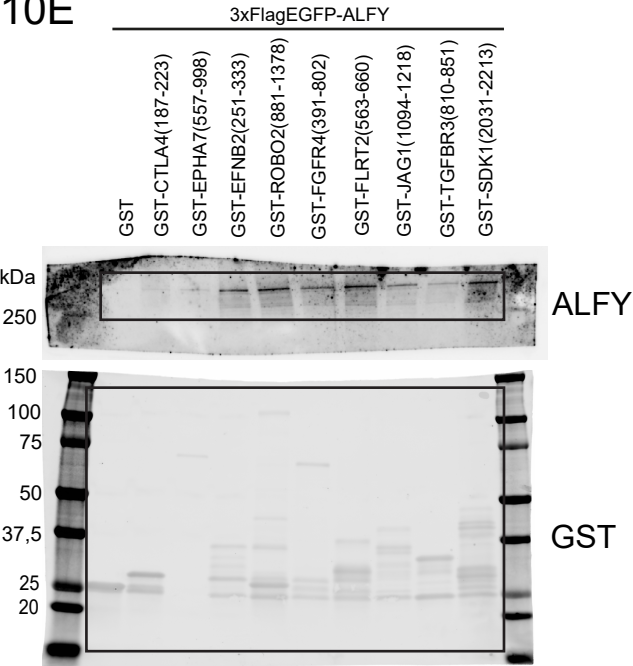

Supplement: SourceData F10 — is the source file for Fig. 10. [file JCB_202408173_SourceDataF10.pdf]

S4C

5KO clones

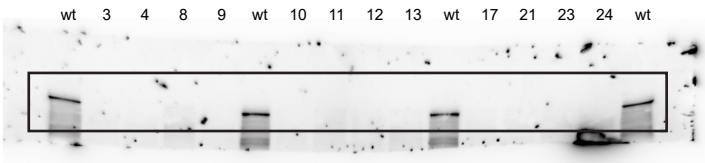

ALFY

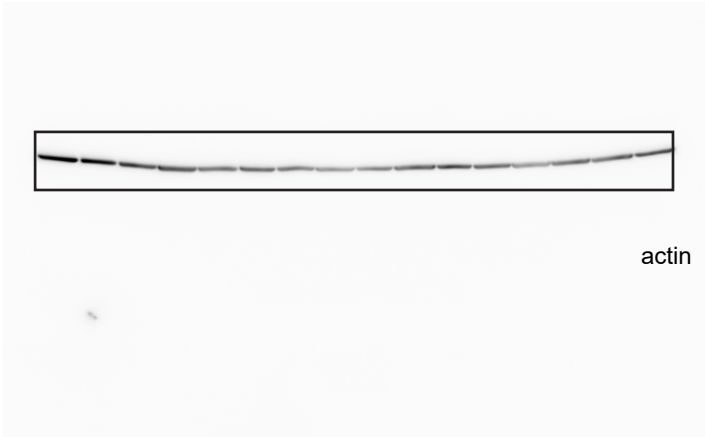

actin

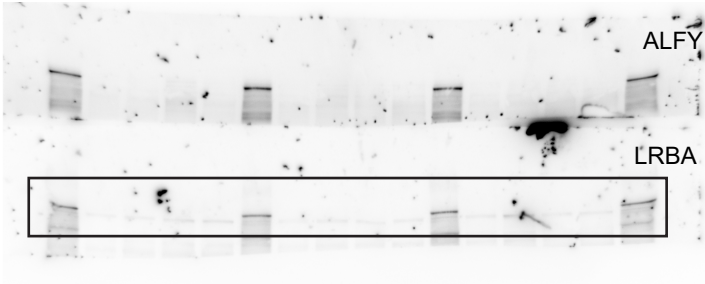

ALFY

LRBA

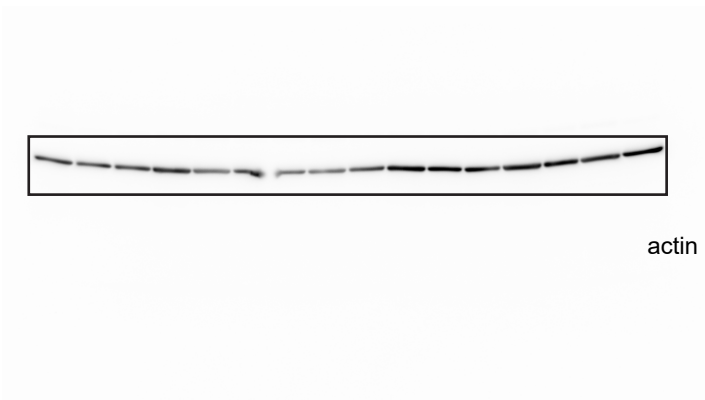

actin

5KO clones

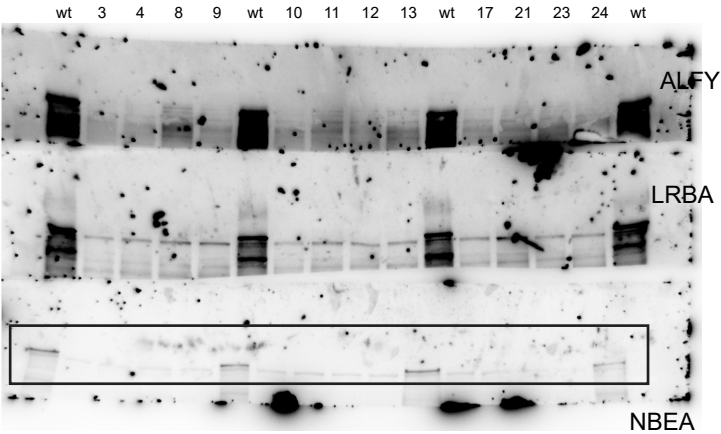

ALFY

LRBA

NBEA

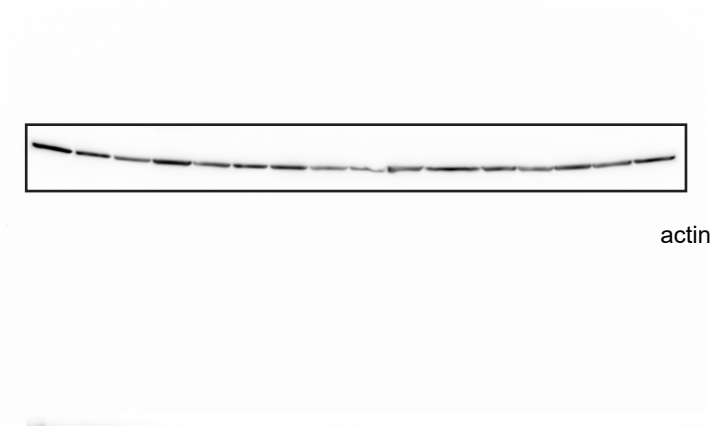

actin

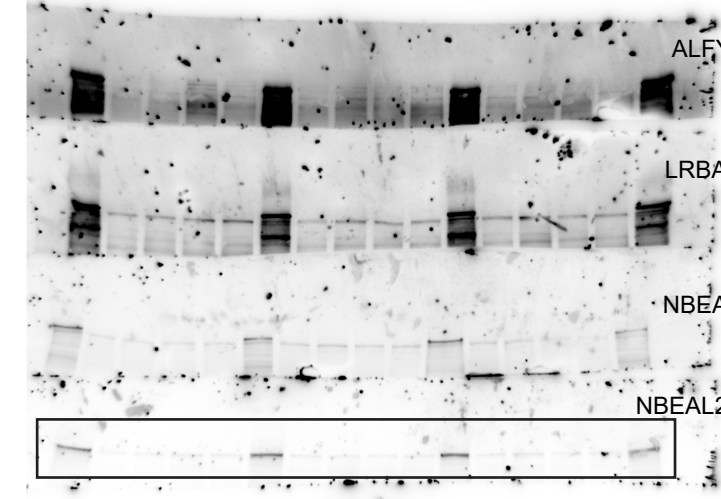

ALFY

LRBA

NBEA

NBEAL2

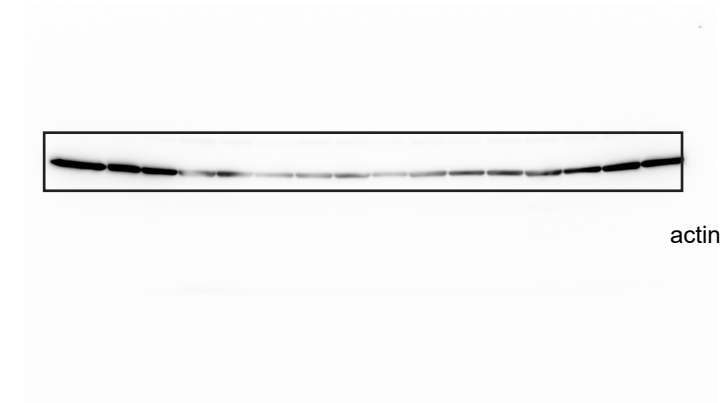

actin

Supplement: SourceData FS4 — is the source file for Fig. S4. [file JCB_202408173_SourceDataFS4.pdf]
